# Coherent X-Ray Diffraction Imaging and Characterization of Strain in Silicon-on-Insulator Nanostructures

**DOI:** 10.1002/adma.201304511

**Published:** 2014-06-23

**Authors:** Gang Xiong, Oussama Moutanabbir, Manfred Reiche, Ross Harder, Ian Robinson

**Affiliations:** 1 London Centre for Nanotechnology, University College LondonLondon, WC1H 0AH, United Kingdom; 2 Department of Engineering Physics, Ecole Polytechnique de MontrealMontreal, Quebec, H3C 3A7, Canada; 3 Max Planck Institute of Microstructure PhysicsWeinberg 2, 06120, Halle (Saale), Germany; 4 Advanced Photon Source, Argonne National LaboratoryArgonne, Illinois, 60439, USA

**Keywords:** coherent X-ray diffraction Imaging, silicon-on-Insulator, strain, ultrathin layer, nanowire

## Abstract

Coherent X-ray diffraction imaging (CDI) has emerged in the last decade as a promising high resolution lens-less imaging approach for the characterization of various samples. It has made significant technical progress through developments in source, algorithm and imaging methodologies thus enabling important scientific breakthroughs in a broad range of disciplines. In this report, we will introduce the principles of forward scattering CDI and Bragg geometry CDI (BCDI), with an emphasis on the latter. BCDI exploits the ultra-high sensitivity of the diffraction pattern to the distortions of crystalline lattice. Its ability of imaging strain on the nanometer scale in three dimensions is highly novel. We will present the latest progress on the application of BCDI in investigating the strain relaxation behavior in nanoscale patterned strained silicon-on-insulator (sSOI) materials, aiming to understand and engineer strain for the design and implementation of new generation semiconductor devices.

## 1 Coherence

Coherence is the property of light responsible for interference effects. Spatial coherence describes the correlation between waves at different points in space, while temporal coherence describes the correlation or predictable relationship between waves observed at different moments in time, or along the direction of a beam. For classical light sources, such as a lamp, a star or synchrotron x-rays, photons are spontaneously emitted in a chaotic way, both spatially and temporally. Spatial coherence can be obtained by applying spatial filter, such as slits, or pinhole, while temporal coherence can be achieved by extracting a small wavelength band using a bandpass frequency filter such as a monochromator. A direct consequence of applying these filters to obtain high degree of coherence is the massive loss of intensity, however with the high-brilliance X-ray sources available in the third generation synchrotron sources, the cost in intensity is now affordable and coherent or partially coherent beams with low divergence can be applied to a wide range of samples. The small divergence also allows most theoretical treatments to adopt the paraxial approximation and the use of the scalar formulation of diffraction theory, neglecting polarization effects in the scattering. Here, some basic concepts related to the coherence properties of a third generation synchrotron source will be introduced to motivate CDI techniques, their recent development, and applications.

### 1.1 Transverse (or Spatial) Coherence Length

This property of light is due to the source not being ideally point-like and having some lateral extension, which can lead to incoherence in the transversal direction. When looking at a cross section of a beam of light, the length over which the phase is correlated is the transverse coherence length. For example, in the case of Young's double slit experiment, if the transverse coherence length of the entering light wave is smaller than the spacing between the two slits, the resulting pattern on a screen in the far field will no longer be a set of interference fringes with fully developed maxima and zero intensity in the minima as would be when the illumination beam is from a monochromatic point source.^1^–^3^ The visibility of the interference fringes will be reduced, in this extreme case to two-single-slit diffraction patterns. For a source of height *w*, with a distance *R* between the source and the observer, the beam is coherent over 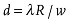, which is defined as the transverse coherence length.

It is worth noting that the transverse coherence length is a property of the source and the beamline optics, and does not depend on the specific diffraction setup. Apart from Young's double slit, a single slit, a waveguide, or a fibre can all be used as a test object to elucidate and deduce the transverse coherent length and obtain the same result.^4^–^6^ The above definition can be extended to rectangular source with horizontal width *w_h_* and vertical width *w_v_* to yield the corresponding transverse coherence lengths 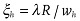 and 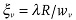.^7^,^8^ The limit 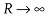 or 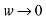 indicate the source being fully coherent with an infinitely large coherence length. 3^rd^ generation synchrotron sources typically have a source size of 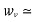
10∼50 μm and 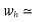 100∼500 μm. At an energy of 9 keV and a typical distance of 30 m from the source, the transverse coherence length are in the ranges of 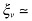
20∼120 μm and 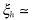
3∼12 μm. For some beamlines which are dedicated for coherent diffraction such as the I13 at Diamond Light Source, with a source-sample distance up to 220 m, the coherence length are larger: 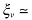
500 μm and 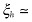
30 μm.

### 1.2 Longitudinal (or Temporal) Coherence Length

The temporal coherence defines the degree of coherence of the light source along its propagation direction and relates to the beam monochromaticity. The temporal coherent length is defined as the propagation distance over which two wavefronts, one with wavelength *λ* and the other with a slightly different wavelength 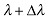, simultaneously departing from a point source are in antiphase. Therefore 
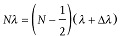
, and one can deduce this distance 
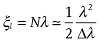
.

The longitudinal coherence length is inversely proportional to the bandwidth. For the (111) reflection of a Si crystal monochromator, 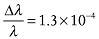. At the energy of 9 keV, the corresponding longitudinal coherence length 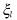
is around 500 nm, much smaller than the transverse coherence length.

The longitudinal coherence length couples to the optical path length difference (OPLD) of the X-rays through the sample. When the OPLD is smaller than 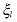 the sample is said to be in the coherent limit and meets the required conditions for coherent diffraction measurements. In the transverse directions, to achieve a full coherent diffraction the sample size should be smaller than the transverse coherence length, or a pinhole of size smaller than the transverse coherence length can be placed just in front of the sample.

## 2 Coherent X-Ray Diffraction Imaging (CDI)

### 2.1 Fundamental Concepts of Lens-Less Imaging

**Figure 1:**
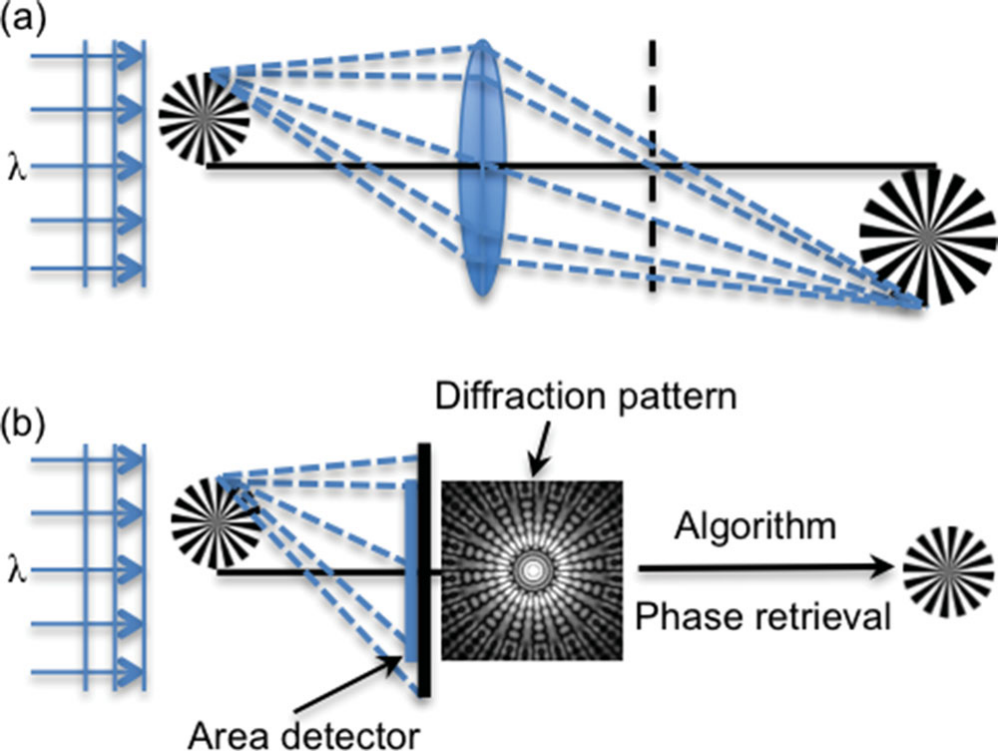
Schematic views of (a) a lens-based imaging system and (b) a lens-less imaging system where the active area of the detector is highlighted with the blue colour. For lens-less imaging, the maximum spatial frequency collected in the diffraction pattern in principle determines the reconstruction resolution.

According to the Huygens principle, every point of an object to which a luminous disturbance reaches becomes a source of a spherical wave. The sum of these secondary waves determines the form of the wave at any subsequent time. Along the propagation direction, a short distance away from the illuminated object, the secondary waves emitted from all object points will interfere and result in scattering patterns in which the object's details are encoded. Different imaging systems can be applied to retrieve the object's details. A lens-based imaging system would typically make use of a lens to reconstruct the object image, as shown in **Figure** **1**a. The lens refocuses the scattered waves with their associated phases to build the image. Lens imaging techniques can be applied to visible light, X-rays and electrons. In such a system, the resolution is mostly limited by the lens aberrations. In comparison, Figure 1b shows schematically a lensless imaging system in which the scattered beam propagates and produces a scattering pattern downstream whose intensity is then collected by a 2D detector. This recorded pattern is then used to reconstruct the object details via an iterative feedback algorithm. Effectively, the objective lens in a typical microscope imaging system is replaced with the algorithm software to convert the scattering pattern from the reciprocal space into a real space image.^9^

Because the detector can only record intensities, the phase information of the scattered complex-valued waves is lost during the measurement. Applying a simple Fourier transform algorithm to the measured intensity alone is insufficient for retrieving the image, giving rise to a “phase problem”. Some constraints need to be applied for both the measurement and sample image in the retrieval algorithms, and will be discussed further in the later sections. The advantage in using lensless imaging techniques is that the final image is aberration–free and resolution is only fundamentally limited by the extent of the diffraction and dose. As schematically shown in Figure 1b, the limit is on the maximum spatial frequency collected in the diffraction pattern which in principle determines the reconstruction resolution. Practically it depends on wavelength, numerical aperture size, photon noise, and radiation damage to the sample.

**Figure 2:**
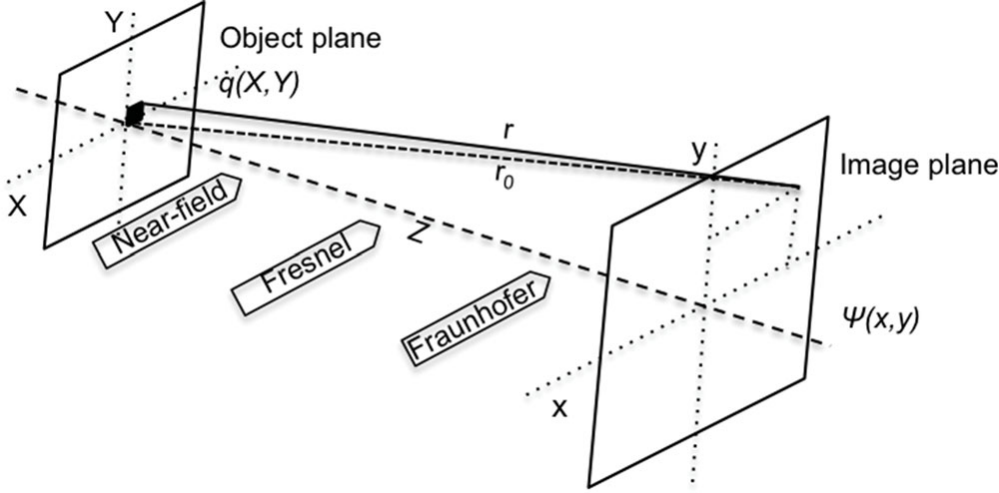
Sketch of three different imaging regions in lens-less imaging system defined as a function of the Fresnel distance 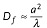 where *a* is the sample size and *λ* is the wavelength.

If the distance between source and sample is *Z_0_*, sample and detector is *Z*, the defocusing distance *Z*_d_ and the magnification factor *M* are then defined as 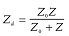, 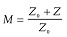, respectively. In lensless imaging, three different imaging regions can be exploited as a function of the Fresnel distance 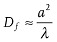 where *a* is the sample size and *λ* is the wavelength, as shown in **Figure** **2**.

In the contact or near-contact regime (*Z*_d_ << *D*_f_), the detector is placed directly behind the sample, the contrast arises as an edge-enhanced image and the sharp edges of the sample appear as characteristic fringes of oscillating intensity.

As the detector moves further away from the sample, interferences build up and the intensity distribution starts to lose resemblance with the original sample. The next regime to enter is the Fresnel diffraction regime (*Z*_d_∼*D*_f_), it can also be called “holographic regime” as the interference pattern is formed by a large number of alternating fringes more and more closed spaced. In this regime, the diffracted amplitude can be approximated as:

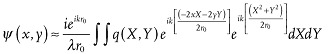
1where 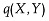 is the complex sample transmission function, with an amplitude due to absorption and a phase due to the shift of the wave propagating through the sample. The third exponential term accounts for the spherical curvature of the wavefront.

Further increase the distance between the detector and the sample, when (*Z*_d_ >>*D*_f_), the curvature of the wavefront becomes negligible and the third exponential term in Equation 1 becomes unity. The amplitude distribution 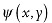 does not change shape with the distance. This defines the Fraunhofer diffraction regime. In this regime, the diffracted amplitude 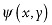 is simply the Fourier transform of 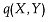.

### 2.2 Phase Retrieval

In a coherent diffraction imaging (CDI) measurement, as the detector can only collect the square modulus of the complex wavefields diffracted from the sample, and any associated phase information is lost. To reconstruct the image of the sample from its diffraction pattern (either Fresnel or Fraunhofer regime), it is necessary to retrieve the phase information from the measured intensity. The phase retrieval algorithms used are primarily based on the iterative method of Gerchberg and Saxton, which was initially developed for the electron microscope imaging case where two corresponding sets of measurements, the magnitudes of an image and its diffraction pattern, are available.^10^ With the computational tool of fast Fourier transformation (FFT) switching back and forth between the two measurement spaces, the complete wave function including both the amplitudes and phases can be determined from intensity recorded in the two planes.

The essence of the Gerchberg-Saxton algorithm lies in the fact of oversampling in diffraction space, which implies sampling the object's density in parts of real space where it is known to be zero. The necessity of oversampling can be illustrated for one dimension case as follows.^3^

In the CDI case, the detector measures only the scattered intensity |F_0_(*u*)|^2^, and the back-transforming results in

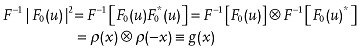
2where 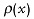 is the sample density distribution function and is defined as 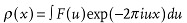. The convolution 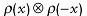 is known as the autocorrelation function of the density. For a sample with non-zero density over a distance *a*, it can be seen from the definition that its autocorrelation function is non-zero over an interval *w* equal to twice the object size *a*. According to Nyquist-Shannon sampling theorem, for a one dimensional function 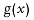 which is non-zero within an interval *w* to be fully reconstructed, its Fourier transform 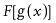 has to be sampled at a spacing no greater 1/*w*. Therefore in the case here, to properly reconstruct the sample's autocorrelation function, one should measure its Fourier transform (
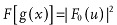
), the scattered intensity, at a spacing 1/*w* = 1/(2*a*). The 1/(2*a*) spacing represents an oversampling of the object's reciprocal space by a factor of two. In the object's real space, the sampling range corresponds to a distance of twice the object size. Since one knows that the object has zero density outside its boundary, this knowledge allows the support constraint to be applied in the real space for solving the phase problem.

The above analysis for oversampling can be extended to higher dimensions. It is suggested that the oversampling by a factor of two refers to the corresponding multi-dimensional space, not to each dimension separately.^11^ Typically, for three dimensional case the oversampling intervals along each axis can be 
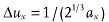
, 
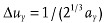
, and 
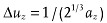
, where *a_x_*, *a_y_* and *a_z_* are the sample's dimensions along the x y and z axes, and each of the three directions has the same oversampling ratio 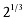.

An oversampling ratio larger than two does not provide any further information.^11^ It results in a larger no-density region and renders the phasing algorithm equations over-determined, therefore could yield a faster reconstruction. However, a larger oversampling ratio requires larger sample-detector distance and results in lower signal-to-noise ratio. In our experiments, we generally make sure that along each axis the oversampling ratio is no less than 3 in order to achieve rapid convergence of the reconstruction.

In the CDI case, there is only one set of measurement accomplished in the diffraction space and no corresponding measurement done in the direct space. Upgraded algorithms based on the Gerchberg-Saxton method have been developed, among them the Error Reductions (ER) and Hybrid input-output (HIO) have been widely applied for phase retrieval in CDI.^12^–^15^ The general principles and procedure of the ER and HIO are described as follows.

**Figure 3:**
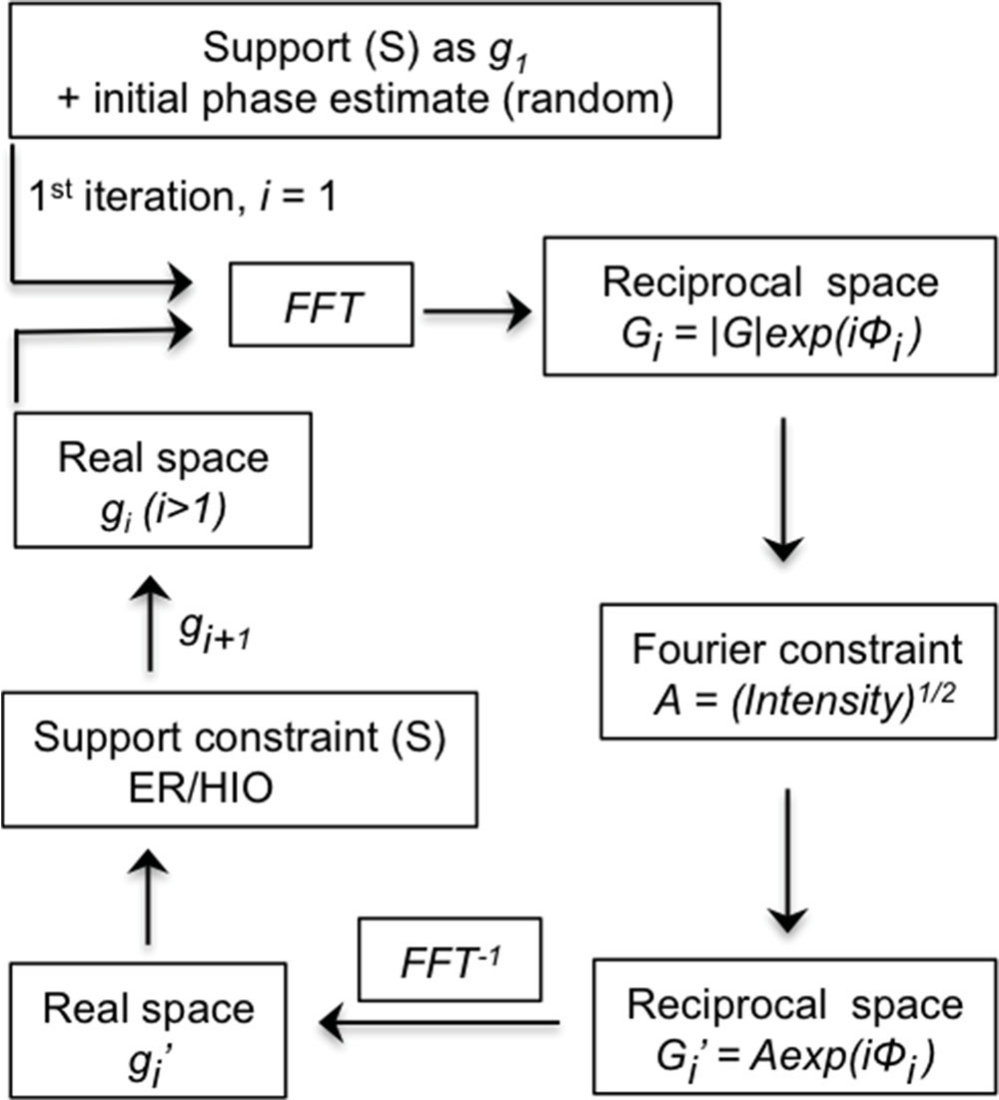
Process flow for a typical phase retrieval algorithm.

As there is no measurement in direct space in CDI, one has to have some prior knowledge of the shape of the object and encode it in a compact support constraint, to aid the phase retrieval algorithm to converge.^16^–^18^ This support constraint can be defined as half the size of the density autocorrelation function 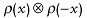. Inside the support region the electron density of the retrieved image is allowed to be non-zero and outside the support the density is padded with zeros. The phase retrieval then proceeds as shown schematically in **Figure** **3**. It begins by assigning a set of random phases to the support. Fast Fourier transformation of the support with its random phases to the reciprocal space yields a diffraction pattern that will be compared with the measured one. After replacing the calculated diffraction intensity by the measured ones, an inverse Fourier transformation (FFT^−1^) is performed and a complex density distribution in real space can be obtained, which generally will have some non-zero values in the area outside the support. One then assigns the density to zero for the part outside the support (for the ER algorithm case) and keeps the part inside the support the same as calculated, and Fourier transforms to reciprocal space again. This iterative process is repeated until convergence is achieved, which means both the Fourier modulus constraints and the support constraint are satisfied. For a convergent solution the error metric 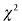, defined as the difference between measured and retrieved intensity 
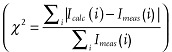
, typically reaches a value smaller than 10^−3^. Here *i* being the index of a given pixel in the computational array, indicates that the calculations are done on a per pixel basis.

The ER algorithm always minimizes the error between the iteration calculation and measurement, thus leaving no access to escape the local minima of the error metric therefore often suffers from “stagnation”. Typical stagnated solutions include the sum of the correct image and its centro-symmetrical inversion, lateral transformation of the object, complex conjugation, spatial inversion etc. The HIO algorithm has been proposed to avoid stagnation, by using a modified real space constraint.^13^,^19^ Instead of setting the estimated object equal zero outside the support region, it takes into account the estimates of the previous iteration using an feedback coefficient β. ER and HIO are shown here in the following equation:

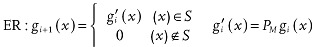
3

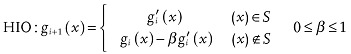
4Where *g_i_*(*x*) is the output of the *i*-th iteration, 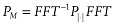 is the projection operator, in which the inverse and direct Fourier transformation are represented by 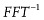 and 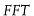, respectively. The HIO algorithm has the effect of damping the regions where the amplitude should converge to zero, and provides the real space constraint that drives the algorithm towards convergence. The feedback component (the second condition in Equation 4) allows this algorithm to emerge from local minima of the invariant error metric and thus avoid stagnation. It essentially controls the convergence rate of the iterations as well. The feedback coefficient β is typically set between 0.6∼0.9.^13^,^19^,^20^

It was proposed that the combination of HIO and ER outperform either of the algorithms separately, and is particularly effective in avoiding stagnation and achieving rapid convergence. The heart of this mixture of algorithms includes a number of cycles of iterations, in which one cycle typically consists of a few tens of iterations of the HIO algorithm followed by 5 to 10 iterations of the ER algorithm.^19^ This procedure has become the workhorse of phase retrieval in coherent diffraction experiments. Recently, a guided-HIO (GHIO) approach has been applied for improving the uniqueness of a reconstructed image.^21^ In GHIO, multiple sequences of phase retrieval are started from random distributions and the resulting images from each are combined together periodically during the iterations, in a variety of ways, to converge to a single solution. A number of other improved approaches have been proposed as well, including the difference map, saddle-point optimization, hybrid reflection projection, relaxed averaged alternating reflections, charge flipping and matrix completion.^22^–^28^

The support is also an important factor for the phase retrieval, as it will significantly influence how the electron density of the image is modified at each iteration. A loose support can result in non-unique solutions to the reconstruction.^18^ Providing a tight support for the phase retrieval requires a *priori* knowledge of the object which is not always available. With this in mind, a formulation called “shrinkwrap” has been developed, which allows for the support size to be dynamically determined as the iterations of the algorithm proceed.^29^ In a ‘shrinkwrap’ reconstruction, the first support can be defined from the autocorrelation function of the object, which is readily obtained by Fourier transformation of the diffraction pattern. Although both the correct object density and its centro-symmetric inversion fit within this initial support, the inversion symmetry would progressively be lost as the algorithm converges. After a certain number of iterations of the algorithm (HIO/ER), the current reconstructed object is convolved with a Gaussian of width *σ* to find the new support. The next round of iterations can then be launched with the new support. This process continues and the Gaussian width *σ* is set to reduce gradually for the support updating in order to eventually achieve a stable solution.

### 2.3 Forward Coherent Diffraction Imaging

**Figure 4:**
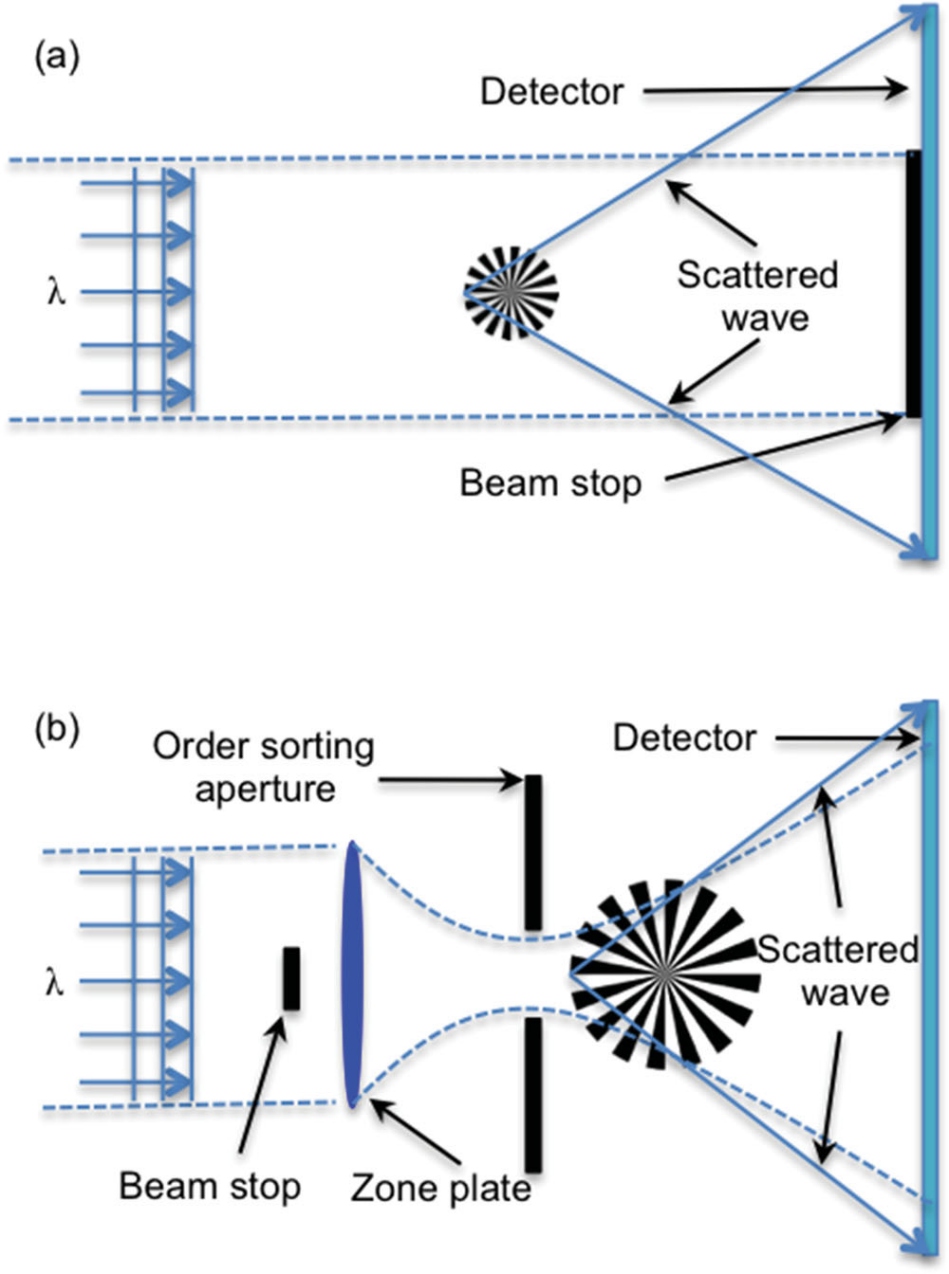
Experimental configuration for (a) plane-wave forward scattering CDI, in which a coherent beam illuminates the whole sample; and (b) Fresnel CDI in which a coherent phase-curved beam generated with Fresnel zone plate illuminates either a whole sample or part of a sample.

The element geometry for forward scattering CDI is fairly simple, in which an isolated object is illuminated with a highly coherent beam of X-rays and the diffraction pattern produced by the sample is measured in the far field, as shown in **Figure** **4**a. Diffraction from an isolated object is very weak in practice, so it is necessary to introduce a beam stop to prevent the direct beam from damaging the detector and to facilitate the dynamic range required to properly measure the diffracted signal. The beam stop effectively prevents the measurement of the intensity at the small diffracted angles.

The first 2D image was recovered by using forward scattering CDI in 1999.^30^ A collection of gold dots, each 100 nm in diameter and 80 nm thick, deposited on a silicon nitride membrane and forming a set of letters. The diffraction patterns were taken by illuminating the sample with an incident plane wave of 1.7 nm wavelength. The reconstruction reproduced the electron microscopy image of the sample, with a resolution of 75 nm. The first 3D reconstruction of an image was achieved in 2002 with a resolution of 50 nm.^31^ The reconstruction was carried out using a series of 2D diffraction patterns recorded from the noncrystalline nanostructured Ni sample. The 3D reconstruction was achieved by using a full range of projections later, in which the iterative process was applied directly to the 3D set of diffraction data and full tomographic images were obtained.^32^ To increase the coherent flux, refractive lens and Kirkpatrik-Baez (KB) mirror systems can be used to illuminate radiation-hard samples, and higher spatial resolution can be achieved.^33^,^34^

Forward scattering CDI can be applied to image functional material samples.^35^,^36^ A typical example shows the first experimental high-resolution view inside a ceramic nano-foam, which reveals a structure consisting of nodes connected by thin beams.^35^ This internal structure can be used for explaining the mechanical properties of the nano-foam and is shown being consistent with a diffusion-limited cluster aggregation model.

Forward scattering CDI can also be realized by placing a suitable optical element (either Fresnel zone plates or waveguides) to focus the incident beam to a virtual point-like source at a position very close to the sample, as shown in Figure 4b.^37^–^39^ This technique is referred to as Fresnel CDI, in which the very small source-sample distance results in spherical waves illuminating the sample and a magnified image is measured on the detector. This image uniquely defines the phase to within a constant and physically meaningless offset. The exit surface wave (ESW) at the sample can be represented as:

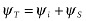
5where 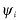 and 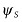 are the illumination through beam and the scattered beam, respectively. As the wave at the detector is related to the ESW at the sample by free space propagation, at the detector plane 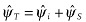 are simply the Fourier transform of the corresponding items in Equation 5. The intensity measured at the detector can be represented as:

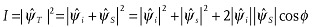
6

As the wavefield illuminating the sample (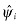) is defined and known, one can see from Equation 6 that the phase of the wave exiting the sample can be retrieved from the measured intensity. Fourier transformation based iterative algorithms have been developed for phase retrieval in Fresnel CDI.^40^–^42^ It was further demonstrated that a finite, diverging incident beam can be used to define the boundary of an extended sample, giving rise to a finite wave exiting the sample.^43^ Therefore any part of an extended sample can be imaged, removing the limitation of the sample being finite in extent.

Although unavoidably introducing some experimental complexities as a consequence of producing the incident curved wave, there are some important benefits in using Fresnel CDI. The presence of a low-resolution image of the object scattering function and the ability to characterize the incident beam from an independent measurement help facilitating the phase retrieval process and result in a faster convergence. Comparing with plane wave illumination, where using a beam stop for the detector is necessary and can results in a loss of low spatial frequency information, Fresnel CDI provide the possibility to measure easily the diffracted intensity at low-*q*. Using this method, buried structures in a semiconductor integrated circuit have been imaged.^44^ Works are under way to develop 3D tomography based on Fresnel CDI.^45^,^46^

Another interesting development is to carry out the CDI measurement with shifting illumination, which is termed as ptychography. Traditional forward scattering CDI has some limitations, including the restriction of samples being finite-size and the difficulty of convergence of the phasing algorithm. Apart from Fresnel CDI, ptychography is another technique may be used to address these limitations. The idea of ptychography was originally proposed in the field of electron microscopy.^47^ In a ptychography experiment, the sample is moved so that the beam illuminates different parts of the sample at each step, but with significant overlapping (60∼90%) between illumination steps. The ptychographical iterative engine (PIE) algorithm has been developed, which utilizes both the redundant information from the overlapping illumination areas on the sample and the iterative phase retrieval algorithms for the CDI, to reconstruct density distribution without the requirement of the sample being confined to a finite size smaller than the beam.^48^,^49^ The convergence problem associated with stagnation in local minima and non-uniqueness of the solution are overcome as well. Another advantage of ptychography is that it can be used as a tool for wavefront characterization of the focused beam due to the redundant amount of data collected.^50^,^51^ To simultaneously retrieve the beam and the sample complex density function results in better reconstruction. Ptychography is a powerful tool for imaging extended samples that cannot be isolated, for example, biological tissues.^52^

### 2.4 Bragg Coherent Diffraction Imaging

Coherent diffraction imaging in the Bragg geometry (BCDI) has recently been developed as a tool for studying nanocrystals, and has become a productive application of CDI.^53^–^56^ BCDI utilizing the exquisite sensitivity of the X-rays to the distortions of crystalline lattice, its ability of imaging strain on the nanometer scale in three dimensions is highly novel. BCDI recovers the complex electron density of the samples with picometer sensitivity and is sensitive to lattice distortion within nanocrystals. The ‘picometer sensitivity’ refers to strain sensitivity much finer than the atomic spacing. The spatial resolution of the retrieved image itself is around 20 nm in these experiments. The ability to achieve picometer sensitivity from a lower resolution measurement is due to the fact that the strain fields are long-ranged, therefore atomic level defects, such as dislocation loops, can be identified by their strain signatures. In this section, we will introduce the fundamental principles and typical experiment setup of BCDI, as well as the recent progress on the method development.

**Figure 5:**
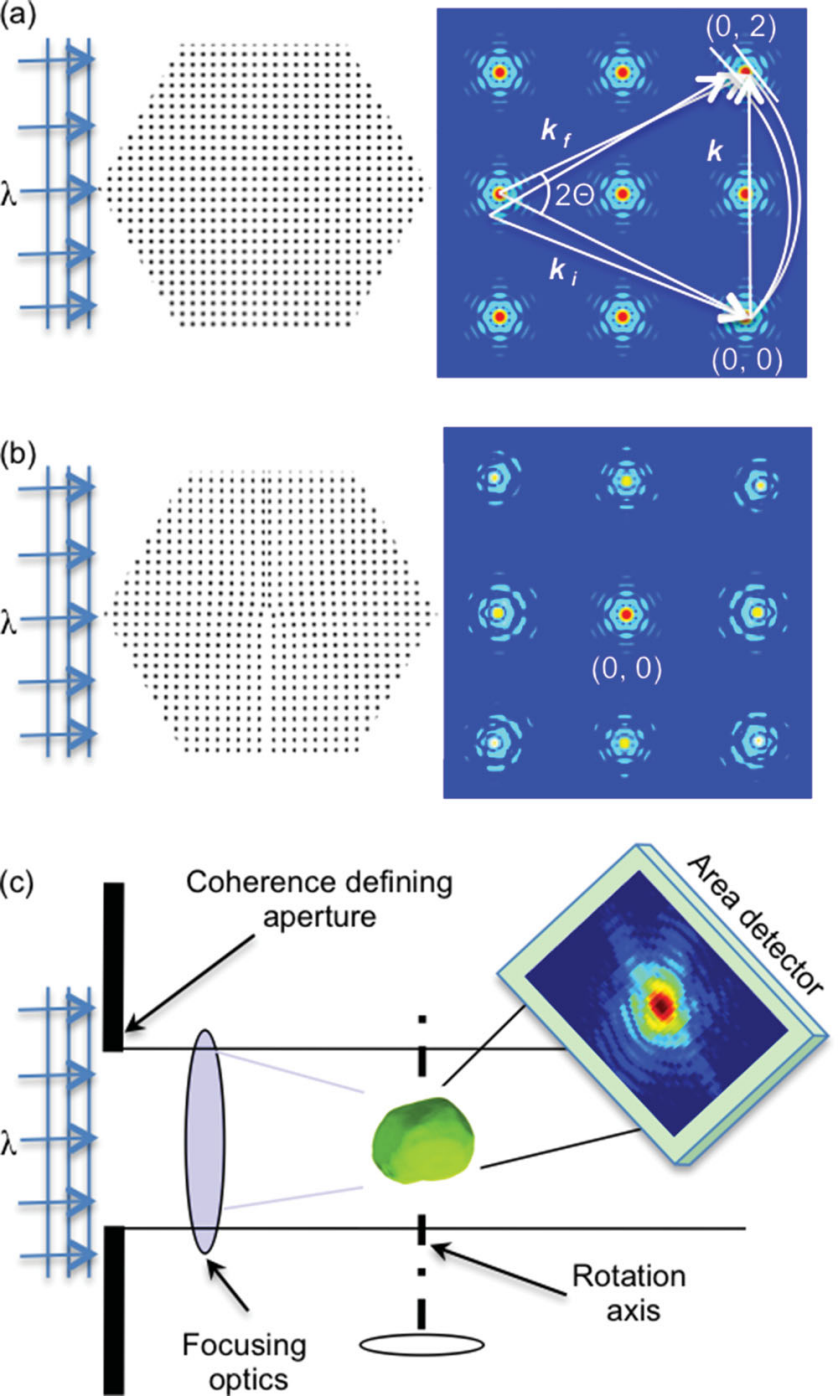
(a) Schematic view of a hexagonal-shaped crystal, where the dots correspond to lattice points (left); and the corresponding diffraction pattern in reciprocal space (right). Also shown here are the incoming (k_i_) and outgoing (k_f_) wave-vectors sketched according to the Ewald construction. As the crystal is rotated the diffraction patterns are collected by the detector (shown as a straight line perpendicular to the k_f_ direction). (b) A crystal with the same size and shape as the one in (a), but with an edge dislocation inside the crystal (left); and the corresponding diffraction pattern in reciprocal space (right). (c) Typical experimental setup for Bragg CDI.

The diffraction pattern of an infinite crystal is the product of the reciprocal lattice and the molecular transform. For a finite crystal, however, the difference is that the diffracted pattern is convolved with the Fourier transform of the crystal shape. The diffraction pattern is the standard crystalline diffraction pattern with a distribution of the intensity at the location of the Bragg diffraction spot. **Figure** **5**a shows a 2D crystal with hexagonal shape on the left (where the dots correspond to lattice points) and its simulated diffraction pattern in reciprocal space on the right. As explained above, the finite size of the crystal results in the extension of the intensity distribution at the Bragg peaks. Furthermore, as an ideal finite crystal with a zero displacement field, the extended intensity distributions are locally centro-symmetric.

Any non-zero displacement field of the atoms in the crystal from their ideal lattice positions (strained crystal), however, will lead to asymmetric intensity distribution at the Bragg spots. Figure 5b shows, on the right, the simulated diffraction pattern for the same crystal as in Figure 5a but with an edge dislocation formed by inserting an extra layer of atoms in the top half of the crystal (Figure 5b on the left). The diffraction intensity distributions around the Bragg spots are no longer symmetrical due to the non-zero displacement field inside the crystal caused by the edge dislocation defect. Except the distribution around the (0, 0) Bragg peak, it is only sensitive to the shape of the crystal and remains unchanged.

To describe the scattering from a strained crystal, the displacement field of the atoms from their ideal positions can be represented as:

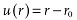
7where *r* is the position of the atom in the crystal and *r_0_* the ideal position of atom in the crystal, respectively.

For coherent and monochromatic scattering, with in the kinematic approximation the scattering amplitude is given by

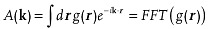
8where **k = k_f_** −**k_*i*_** is the scattering vector (**k_*i*_** incoming and **k_*f*_** outgoing wave-vectors, 
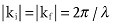
, *λ* is the wavelength), *g*(*r*) is the complex-valued electron density of the crystal and given by

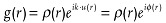
9where *ρ*(r) is the 3D scattering factor distribution within the crystal, 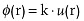 is the phase factor.

The experimentally measured intensity *I(**k**)* is related to the scattering amplitude *A(**k**)* by^57^

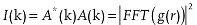
10

When the transverse coherence length of the incident beam is larger than the dimension of the crystal sample and the diffraction pattern is adequately oversampled, one can reconstruct *g*(r), the complex-valued electron density of the crystal, from the measured intensity distribution around the Bragg spot using the phase retrieval approaches discussed in Section 2.2. And the reconstructed complex electron density comprises both the amplitude, corresponding to the crystal morphology, and the phase, which is related to the displacement field (A phase of 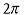 represents a displacement from the ideal lattice position with a distance equal to the lattice spacing in the scattering vector direction). The strain is related to the displacement field by^58^

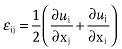
11where *x_i_* is the spatial coordinate in the orthogonal direction *i*. BCDI is therefore potentially a powerful tool in investigating strain related phenomena, including crystal defects, strain relaxations in semiconductor devices, and phase transition etc. In this report, as will be shown in next section, BCDI's ultra-high sensitivity to lattice strain is exploited to investigate the strain distribution in sSOI structures, aiming to understand and engineer strain properties for design and implementation semiconductor devices.

In the Bragg reflection vicinity there is another advantage with respect to the forward scattering CDI: the 3D reciprocal space mapping becomes much easier.^54^ A slight rotation of the crystal have the effect of rapidly rocking the diffraction spot through the diffraction condition. Figure 5a, on the right, illustrates the case for the measurement at the vicinity of reflection (0,2). The incoming (**k_i_**) and outgoing (**k_*f*_**) wave-vectors are sketched according to the Ewald construction. During the measurement, the sample is rotated through the Bragg condition for acquiring a sequence of nearly parallel patterns, which are shown as straight lines perpendicular to the outgoing wave-vector. This procedure is equivalent to shifting the detector perpendicular to the Ewald sphere in reciprocal space. Because the diffraction pattern surrounds a reciprocal lattice point is located far from the origin, the entire intensity distribution spans only a small angular range, which in most cases lies within a 1° sample rotation range. By contrast, the forward-scattering version of the experiment requires a complete 180° rotation. This principle can be extended to 3D reciprocal space, and by using a 2D detector to collect the diffraction slices at each rotation step and stacking them together, a full 3D diffraction pattern can be obtained.

Figure 5c shows schematically a typical BCDI experiment setup. A monochromatic X-ray beam is selected by passing the beam through a Si-(111) double crystal. A set of JJ slits is used to select a coherent beam with a size of a few tens of microns, followed by focusing optics, such as a pair of Kirk-Patrick Baez mirrors or a Fresnel zone plate, to focus the beam down to a range of less than a hundred nanometers to 2 μm. It is worth noting that the focusing optic is used for concentrating the coherent beam and maximizing the flux illuminating on the nanocrystal sample, and does not determine the resolution of the reconstructed image. The single crystal sample, positioned downstream, is then illuminated by the focused beam. Diffraction patterns are gathered by rotating the sample through the Bragg condition in small increments, typically 0.002∼0.01° determined by the oversampling condition. At each step, 2D slices of the diffraction pattern are acquired by the X-ray detector and subsequently collated to form a complete 3D diffraction pattern for reconstruction.

The first successful 3D quantitative phase retrieval was demonstrated on a lead nanocrystal which was crystallized in ultrahigh vacuum from a droplet on a silica substrate.^55^ A 3D image of the density, obtained by inversion of the coherent X-ray diffraction, shows the expected facetted morphology, but in addition the 3D displacement field mapping revealed a real-space phase that is consistent with the three-dimensional evolution of a deformation field arising from the superimposes of interfacial contact force from classical point defects.

As can be seen from Equation 9, the phase factor is the scalar product of the displacement field and the scattering vector, therefore only the component of the displacement field corresponding to its projection in the scattering vector direction can be retrieved from a single diffraction measurement. In order to obtain the full strain tensor in a crystal, it is necessary to measure at least 3 non-coplanar Bragg spots. This has been demonstrated in a recent work, in which a ZnO nanorod was measured at 6 different Bragg peaks.^59^ From each diffraction pattern a 3D map of the displacement field's component along the corresponding scattering vector direction is retrieved. By combining these components together, all three Cartesian components of the displacement field in the nanorod, and in turn the full nine-component strain tensors have been imaged for the nanorod.

Another progress is the addition to the retrieve algorithm of the ability to correct for imperfect coherence in the incident X-ray beam.^60^ The imperfection in coherence can be due to the scatter from window that the beam passes through, or slits open to a size wider than the beam coherent length even though this would allow one to utilize more flux to reduce the measurement time. Partially coherent wavefield illumination can reduce the contrast between the bright and dark regions of the diffraction pattern. In real space, the recovered density amplitude will typically have unphysical density modulations and be less uniform.^61^–^63^ Therefore it is ideal to perform CDI measurement with fully coherent beam. However many CDI experiments use third generation synchrotron or electron sources that can be far from fully coherent.^42^,^64^–^66^ Efforts have been made in adapting the current algorithms to accommodate both spatial and temporal partial coherence.^67^–^70^ In recent developments, modal methods have been introduced which assume that the recorded diffraction is made up of a number of independent modes, with the recorded diffraction being the incoherent sum of each mode's intensity. With this modified modulus constraint, 3D ab initio phasing of partially coherent diffraction patterns has been demonstrated. Image quality can be improved while simultaneously recover the coherence properties of the illuminating wavefield without a *priori* assumptions.^60^

One of the important themes of nanoscience is the emergence of new phases of matter with novel functions at small sizes and size-dependent phase transitions. In BCDI, these behaviors will be detectable as displacement field (phase) variations within the nanocrystals. Strong surface segregation effects during the intermixing of alloy formation can be observed as the complex density change, so as the case for surface vacancy diffusion. With the ability to achieve quantitative 3D imaging of lattice strain on the nanometer scale, BCDI is becoming a powerful technique for the structural characterization of nano materials and devices. A wide range of applications has been exploited based on the BCDI technique, typical examples including the studying of nanocrystals,^71^–^76^ nanowires,^77^–^82^ catalyst microcrystals,^83^,^84^ materials under high pressure,^85^ lattice dynamics^86^,^87^ and examination of the diffusion behaviors inside nanocrystals,^88^ among others,^89^–^98^ by analyzing the structure, defect and strain either in the static or dynamic process. In the following section, we will focus on the progress on the strain distribution investigation in strained-silicon-on-insulator structures, in which the strain plays a critical role in enhancing device performance.

## 3 Strain Distribution in Silicon-on-Insulator (SOI) Structures

### 3.1 Silicon on Insulator

In semiconductor industry, Moore's law predicts that the number of transistors per square inch, and the chip performance will be doubled approximately every 18 months.^99^ This law has proven to be amazingly accurate since 1960, in part because it has been used to guide long-term planning and to set targets for research and development. The exponential scaling trend in the reduction of the feature size in integrated circuits has enabled the microelectronic industry to produce products with impressive increase in computational capability. This relentless progress towards miniaturization has led to a continuous shrinkage in the channel width from a few micrometers in early 1970s to below 10 nm in the current and planned technology nodes.^100^ However, it has indeed taken countless great efforts from engineers and scientists to avoid the realization of many predictions of a near-term end of device scaling in the past. This scaling trend is expected to hit the fundamental limits when the channel width reaches the atomic dimension. New materials or (and) device structures are thus needed to sustain the current rate of progress in device technology.

Silicon-on-insulator (SOI) structures, consisting of a film of single crystalline Si separated by a layer of SiO_2_ (or sapphire, but less often) from the bulk silicon substrate, have been proposed and developed as an alternative substrate to extend the lifetime of the traditional silicon-based metal-oxide-semiconductor field-effect-transistors (MOSFET).^101^,^102^ Since around 2000, commercial applications of SOI have grown rapidly and entered the mainstream of ultra-large scale integration (ULSI) electronic circuits.

The addition of an insulator layer below the device junction layer can greatly reduce the junction capacitance and leakage current, and brings a few striking advantages.^103^–^106^ First, as the features in the microchip keep scaling down and approaching the end of ‘the international technology road map for semiconductors',^100^ applying SOI technology can keep the traditional Si technology going besides creating new horizons for a variety of innovative applications. MOSFETs with gate lengths of 25 nm or less do not perform well when built on bulk Si. The electric field in the transistor channel induced by the gate has to compete with the field from the source and drain regions. When the source and drain are brought into proximity the gate can lose the control of the channel electric carriers. It can not turn off the device and the transistor ceases functioning. These short channel effects (SCE) are reduced or eliminated by going to thin SOI structures as they eliminate most of the leakage paths. And for the same supply voltage, microprocessors run faster in SOI than in bulk Si. Alternatively, it is possible to lower the operating voltage for SOI chips while still keep the clock rate the same as in the bulk Si circuits, thus reduce the power consumption. Devices built on SOI are radiation-hard, as the majority of charges generated by a radiation particle hitting on a Si substrate would be retarded by the buried oxide layer. Other development using SOI include power and high voltage devices, and high temperature circuits etc.^107^

Strained-silicon-on-insulator (sSOI) has emerged as a new variety of SOI materials with enhanced capabilities. It has the same structure sequence as SOI, but with the top layer being a strained single crystalline silicon instead.^108^,^109^ It combines the advantages of SOI technology and that of the strained silicon. Strained silicon is adopted in an attempt to enhance the carrier mobility, as it can improve device performance beyond any benefits from device scaling and the addition of insulator layer below the devices as in SOI. Strain engineering alone has been proved both experimentally and theoretically to enable drive current enhancement of ∼4.5 times for Si pMOSFETs (compressive strain) and ∼2 times for nMOSFETs (tensile strain), without a significant increase in leakage current.^110^,^111^ Since the 65 nm technology node, strain has been introduced to improve the carrier transport in Si-based CMOS devices.

**Figure 6:**
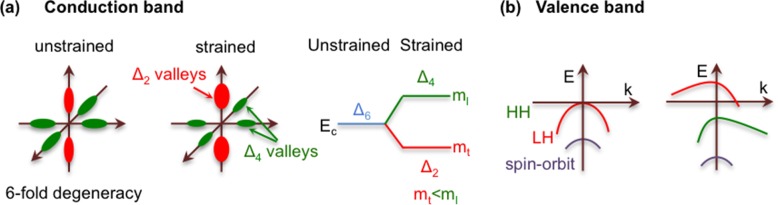
Biaxial tensile strain effects on the conduction and valence bands of silicon (100). (a) In the conduction band, the 6-fold degeneracy is lifted and electrons become repopulated into the lower energy Δ2 sub-band, which causes the average effective mass to decrease and inter-band scattering to reduce, therefore increasing the electron mobility; (b) in the valence band, The hole mobility enhancement is mainly due to the reduction of the phonon scattering caused by the lifting of the 2-fold degeneracy and lowering of the spin-off band.

The use of strain as technology booster relies on its effect on altering silicon band structure. **Figure** **6** shows the biaxial tensile strain (in the *x*-*y* plane) effects on the conduction and valence bands of tensile strained silicon.^112^ In this case, the spacing between the atoms in the plane of the wafer is bigger than it is for regular silicon. For the bulk silicon, there are six degenerate valleys with the minimum energy located near the X point in the conduction band. The tensile strain breaks this degeneracy, and shifts and splits these subbands, causing the energy of the Δ2 subband to shift down and the energy of the Δ4 to shift up. These will result in electrons repopulating from the Δ4 subband to the Δ2 subband, as shown in Figure 6a. The effective mass of electron in Δ2 valley is much smaller than that in the Δ4 valley, and the repopulation into Δ2 has an effect of reducing the average effective mass of the electrons thus increasing the electron mobility.^113^ The phonon scattering rate also changes due to the band splitting. The band splitting causes the decrease of the density of states (DOS), therefore the inter-band scattering rate becomes lower and contributes to the increase of the electron mobility.^114^

In the valence band, for bulk silicon the band minimum is located at the 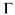 point, where the heavy-hole and light-hole bands are degenerate. As shown in Figure 6b, in contrast with the conduction band, the shape of the sub-bands become changed under strain and this is referred to as “band warping”. The lifting of the degeneracy between light-hole and heavy-hole bands and lowering of the spin-off band, result in the reduction of inter-band and intra-band phonon scatterings, which is the main factor in the hole mobility improvement, rather than the reduction of the effective carrier mass which is the dominant mechanism in the conduction band.^115^

**Figure 7:**
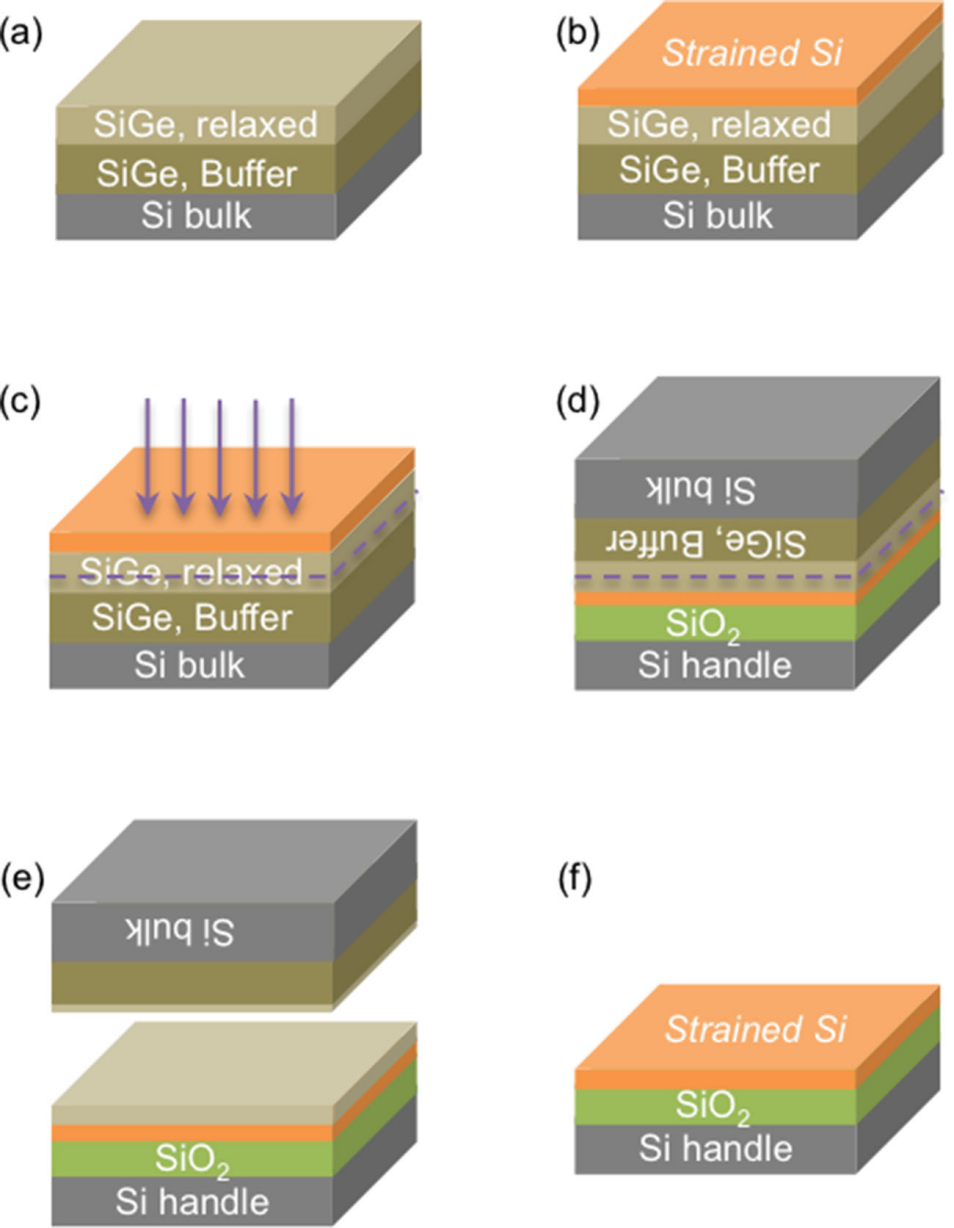
Schematic presentation of the process flow for the fabrication of a sSOI wafer. (a) Growth of the relaxed SiGe substrate; (b) growth of the biaxially tensile strained Si on the SiGe substrate; (c) hydrogen ion implantation into the grown heterostructure; (d) bonding of the hydrogen implanted heterostructure to a SiO_2_/Si substrate; (e) thermal annealing induced layer exfoliation around the hydrogen implantation depth; (f) strained Si layer directly on SiO_2_/Si obtained after the removal of the residual SiGe with selective etch.

Various methods can be used for introducing strains in semiconductor devices during fabrication. Etch-stop nitride and embedding SiGe in the source/drain region are among the most widely used approaches.^116^,^117^ In these cases, the strains are generated locally during transistor processing. This approach, known as a local strain process, and the strains induced are typically uniaxial. sSOI, on the other hand, is used in a global strain process, where the devices are directly built on the strained silicon. The fabrication of sSOI substrate requires technique development on both strained silicon heteroepitaxy and SOI. To obtain tensile strained silicon layer, germanium is an ideal template substrate for the epitaxial growth, as it has the same crystal structure as silicon but with a larger crystal lattice. However in practice, to avoid generating large numbers of crystalline defects, such as misfit and threading dislocations, SiGe alloys are used as the growth template. For example, a SiGe template layer incorporating 20% Ge will usually have a dislocation density below 10^5^ cm^−2^. The lattice mismatch can also be controlled by adjusting the ratio of Si to Ge in the template. **Figure** **7** shows schematically a typical process flow for sSOI fabrication. The first step is to epitaxially grow a SiGe buffer layer on bulk silicon, with the fraction of germanium starting from zero at the bottom and going up to the final value (20%, for example) at the top (Figure 7a). The SiGe template layer is then grown with the same lattice constant as the top of the buffer layer. This template layer has no strain in it therefore is ‘relaxed’, and this allows an optimally tensile strained silicon layer to grow on it subsequently (Figure 7b).

After the growth, hydrogen ion implantation is applied to the obtained heterostructure under ion-cut optimal conditions (Figure 7c).^118^ The implanted wafer is then bonded onto a handle wafer, which consists of a silicon substrate with a SiO_2_ insulator layer on the top deposited by thermal oxidation or in a plasma-enhanced chemical vapor reactor (Figure 7d). Thermal annealing at an intermediate temperature (∼500 °C) induces micro-cracking, which results in exfoliation around the implantation depth (Figure 7e). A strained Si thin layer on an insulator (sSOI) is obtained after the removal of the residual SiGe buffer layer using selective chemical etching (Figure 7f). The thickness of the strained silicon layer is usually in the range of 10∼30 nm. Thicker layers can be obtained by additional homoepitaxial growth without significant relaxation of the strain. For a 0.6% initial strain it has been found that the strain can be preserved during the subsequent homo-epitaxial growth up to a thickness of 60 nm.^119^

### 3.2 Strain Distribution in sSOI Structures

The application of strain engineering in device fabrication raises fundamental questions about the evolution and stability of the strain during different processing steps. It was shown that the strain in sSOI can be maintained during high temperature annealing.^120^ However, the effective strain in semiconductor structures can still be drastically altered due to the integration process, particularly the active region patterning.^121^,^122^ There is strong motivation to understand the fundamental properties of strained Si structures during the different steps of processing, which requires accurate probe of the local strain.

Recent progress in probing the strain distribution in nanostructures includes utilizing Raman spectroscopy,^123^–^125^ transmission electron microscopy (TEM) based techniques,^126^–^128^ and x-ray diffraction based techniques, such as microbeam x-ray diffraction,^129^,^130^ high resolution x-ray diffraction,^131^,^132^ and grazing incidence x-ray diffraction (GIXRD).^133^ Raman spectroscopy provides a fairly good spatial resolution and does not require any specific sample preparation, but it is limited to bare Si structures as the top metallic layer in the device prohibits the laser penetration. The heating of the sample by the laser have to be dealt with carefully in order to avoid any parasitic frequency shift, especially for thin Si layers on SiO_2_, where the thermal conductivity is poor. Therefore a careful experimental protocol is needed.

The latest TEM-based techniques, such as nanobeam diffraction and dark-field holography, enable sub-10 nm resolution in strain mapping.^128^ Nevertheless, the major limitation of these techniques has to do with the relaxation during TEM specimen preparation, which not only requires mechanical simulations for interpreting the data but also results in uncertainties in strain analysis. High resolution X-ray diffraction and GIXRD allow characterizing the strain without special sample preparation. However, as the beam footprint on the sample is in millimeter scale, those techniques integrate and average the reflection intensities of a number of structures. Very small deviations from ideal periodicity can cause the smearing out of any ultrafine fringes.^132^

BCDI with the nature of extreme sensitivity to lattice distortion (strain), high spatial resolution, and the possibility of measuring embedded devices ‘in operando’, has clear advantages compared with aforementioned techniques in characterizing strain distribution in micro- and nanostructured semiconductor devices. BCDI based ptychography has been applied to probe the strain distribution in a semiconductor device prototype.^134^,^135^ A probe beam was focused down to 85 nm and scanned over an epitaxial stressor layer of a SiGe-on-SOI structure with a step size of 25 nm, and 2D slices of diffraction around the (004) SiGe Bragg peak were collected at each step. The reconstruction reveals that the internal strain profile consisted of two competing lattice distortions, one from the rotation of the SOI lattice and the other from the SiGe near-edge film mismatch response. The sum of the two distortions quantitatively account for the asymmetric SiGe lattice slope profile observed in the Bragg ptychography measurements.^135^

Here we present the progress on applying BCDI to study the strain relaxation behaviors due to lithography patterning for individual and multiple sSOI structures.^136^

#### 3.2.1 Strain Relaxation in Individual sSOI Structures

In this work, sSOI wafers consisting of ultrathin strained Si film with a thickness of 20 nm were used for investigation. The strained Si film was epitaxially grown on a Si_0.84_Ge_0.16_ layer. Using direct wafer bonding and ion-cut process as described above, the strained Si layer was then transferred onto a Si wafer with a 200 nm thick SiO_2_ layer in between. The transferred layer is under a biaxial tensile strain of 0.6% as measured by Raman spectroscopy. In the X-ray measurement we compared the (-111) Bragg 2*θ* angles between the sSOI layer before patterning and the unstrained Si, and there is a 0.05° shift between them which is in agreement with the existence of a 0.6% biaxial tensile strain.

**Figure 8:**
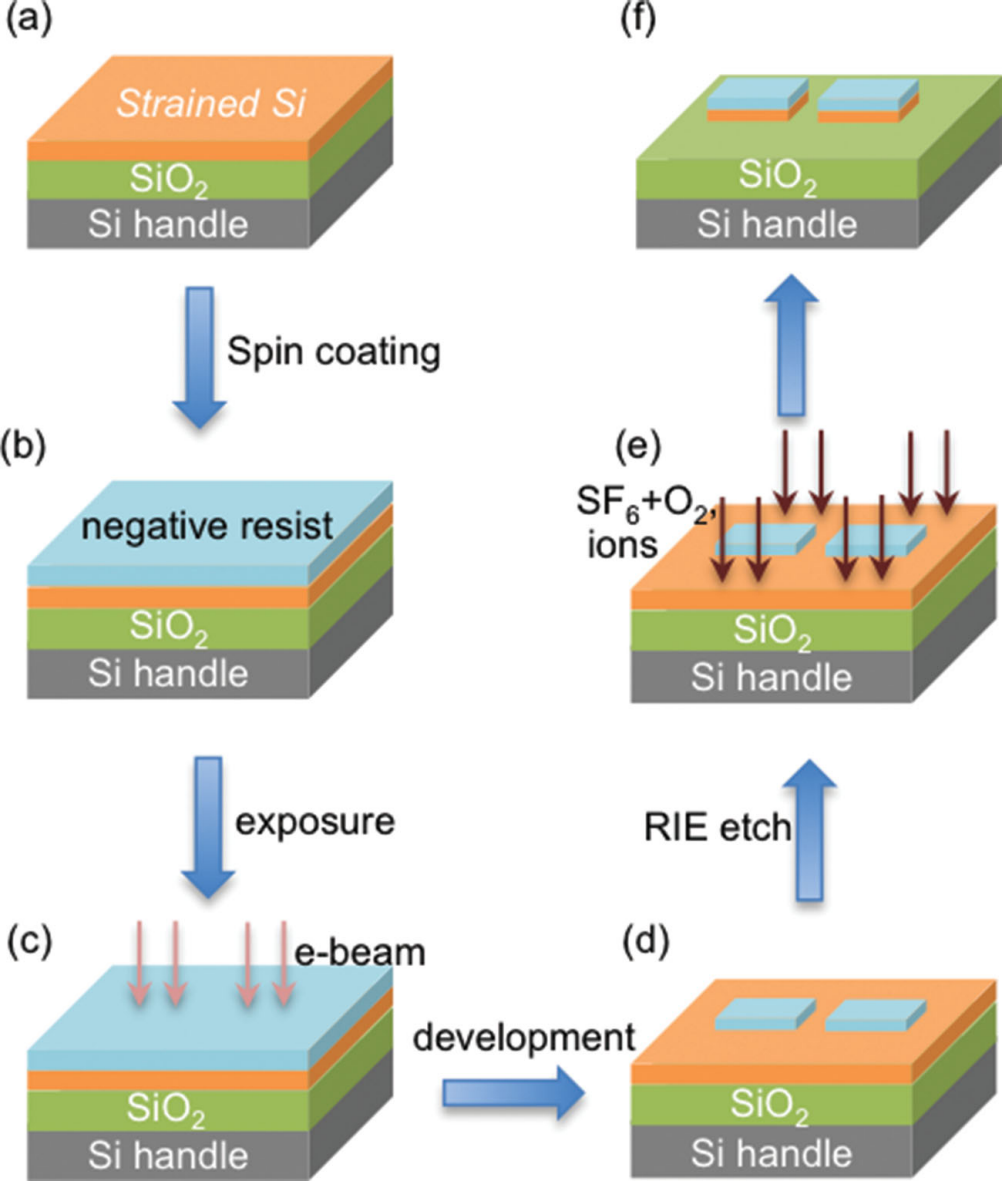
Schematic presentation of the process flow for patterning square strained silicon structures on a sSOI substrate, using E-beam lithography.

Electron-beam-lithography process was applied to pattern the sSOI layer, as schematically shown in **Figure** **8**. A negative resist is spin-coated on the sSOI substrate. Then a square array (20 × 20) of square elements with a lateral dimension of 1 μm, separated by 100 μm, was patterned on the negative resist by electron beam exposure. After development, the areas exposed by electron beam remained on the substrate and reactive ion etching (RIE) was applied to transfer the pattern to the strained layer, leading to array of square strained Si structures on oxide. The patterned islands are aligned along the <110> direction.

**Figure 9:**
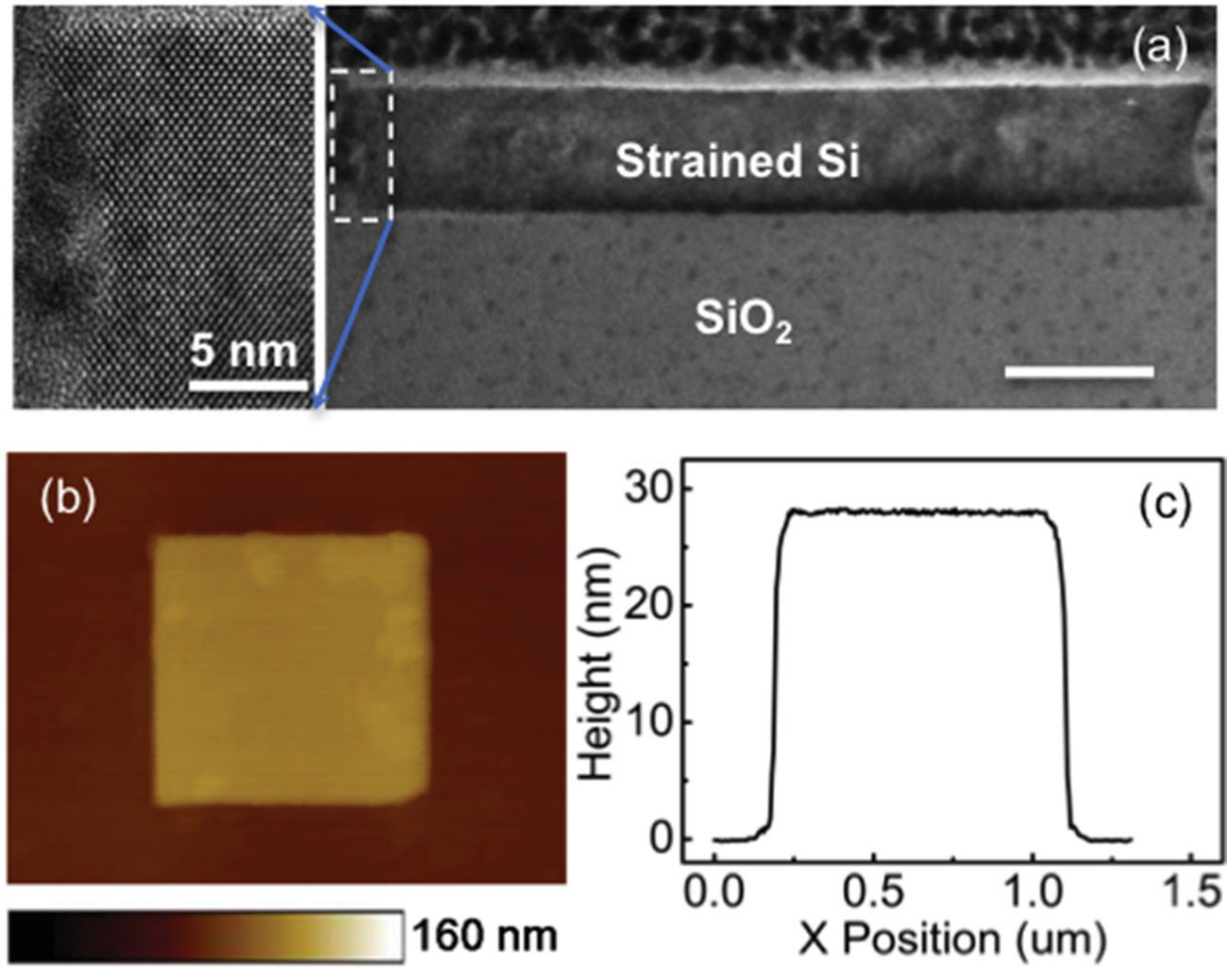
(a) High resolution TEM image of the edge profile of the strained-silicon-on-insulator structure after RIE etch; inset is the zoomed in view; (b) AFM image of a 20 nm thick 1 μm ×1 μm. strained Si structure and (c) the height profile across the center of the structure.

The etching was performed at −60 °C using a mixture of SF_6_ (100 sccm) and O_2_ (5 sccm) with a relatively low power of 40 W. The etch condition is optimized so that the chemical reactivity is dominant and the edges are formed with minimum damages, as confirmed by high resolution TEM investigations, shown in **Figure** **9**a. Figure 9b displays a typical atomic force microscopy (AFM) image of the investigated structures. The square has a lateral size of 950 nm × 950 nm slightly below 1 μm × 1 μm, indicating a small shrinkage of the transferred pattern, which could be due to fluctuations during one of the pattering steps. The cross section profile (Figure 9c) of the structure shows that the island has a height of 28 nm, which means the etching has gone deep enough to cut through the 20 nm thick Si layer and ensured that every square is an isolated structure. As we shall see later, this over-etch also has a significant influence on the strain relaxation of the patterned structure.

**Figure 10:**
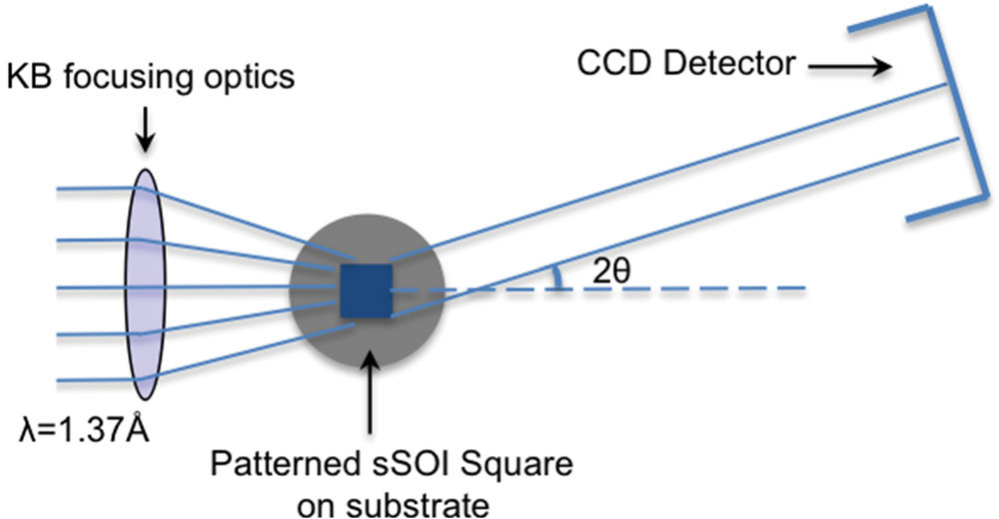
Schematic drawing of experimental geometry. The detector was positioned 1.5 m from the sample, with in-plane angle δ = 21.3°, out-of-plane angle *γ* = 14.6°, Bragg angle 2θ = 25.3° for Si (-111) diffraction.

The BCDI experiments were performed at the Advanced Photon Source, Argonne National Laboratory, Beamline 34-ID-C. **Figure** **10** shows the schematic drawing of the experiment geometry. A 9 keV coherent X-ray beam was focused to about 1.5 μm with Kirkpatrick-Baez mirrors, so the beam size is enough to fully illuminate a single patterned square sSOI structure. Because the large separation (100 μm) between neighboring square elements, and a higher incidence angle (8°), it is ensured that only the diffraction signals from one individual square structure was collected. Diffraction patterns were measured by rotating the sample along the rocking curve of the (–111) Bragg reflection. A CCD detector with 20.0 μm pixel size was positioned 1.5 m downstream of the sample, with in-plane angle *δ* = 21.3°, out-of-plane angle *γ* = 14.6°, and total Bragg angle 2*θ* = 25.3° to collect the 2D diffraction slice for each rotation step. The step size and detector distance are chosen so that the over-sampling ratio is no smaller than 3 along any direction.

**Figure 11:**
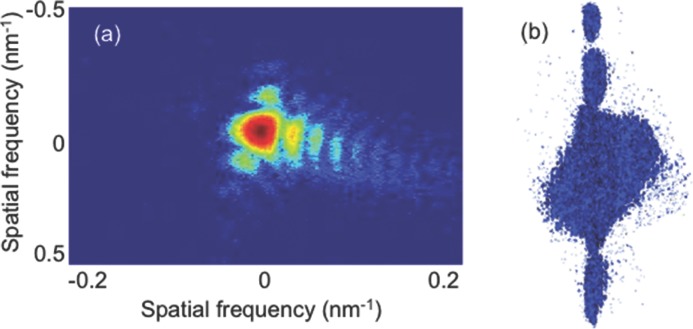
(a) The 2D diffraction frame collected at the centre position of the rocking curve; (b) the 3D diffraction pattern obtained by stacking the collected 2D frames together, projected in the direction perpendicular to the sSOI thickness axis.

**Figure** **11**a is the 2D diffraction pattern at the centre position of the (–111) rocking curve, collected by the CCD detector. Figure 11b is the 3D diffraction pattern obtained by stacking all the collected 2D frames along the rocking curve, with the projection in the direction perpendicular to the sSOI thickness axis. They both present an asymmetrical distribution of the diffraction fringes, which is the characteristic of the investigated structure being strained.

**Figure 12:**
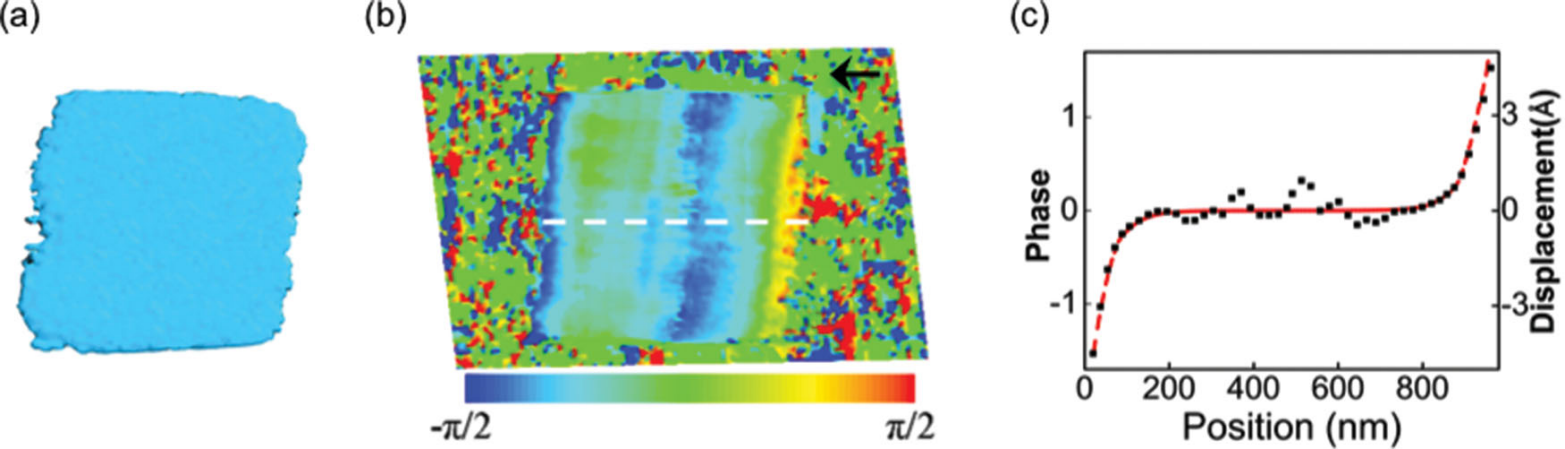
The reconstructed amplitude (a), phase (b), and a cross section plot of the phase variation along the x direction (black square), with the COMSOL-simulated displacement (red dashed line) (c).

The 3D pattern was inverted using a Hybrid Input-Output phase retrieval algorithm with a cuboid-shaped support and a [-*π*/2, *π*/2] phase constraint. **Figure** **12**a and 12b are the reconstructed magnitude and phase of the square structure. Here, a phase of 2*π* represents a displacement from the ideal lattice position with a distance equal to the lattice parameter in the {–111} direction, which is 0.3134 nm. The magnitude shows that the structure has a lateral size of 930∼940 nm, consistent with the AFM measurement. The phase map shown in Figure 12b represents the displacement along the Q-vector direction, which is aligned along the horizontal edges of the square for the (–111) reflection, pointing to the left as shown by the arrow. It can be seen that there are two strong phase stripes near the left and right edges, and the corresponding displacements are relatively compressive and point to the centre. The fact that the edges are contracted is attributed to the formation of free surfaces from the RIE patterning, which leads to relaxation of the initial tensile strain in the film. The relaxation is pronounced in the region within ∼150 nm from the edges and rapidly attenuates towards the center where the initial strain is preserved.

It worth pointing out that the contraction is observed on the vertical edges. As discussed in section 3.1, the displacement component vertical to the scattering vector cannot be detected from (–111) reflection. In the geometry of this experiment, the scattering vector for the (–111) reflection is mostly parallel to the horizontal edges of the sSOI square as shown in Figure 2b, so is less sensitive to the vertical component of the contraction.

According to Rayleigh wave solution of the continuum elasticity equation, the strain surrounding a surface distortion should die off exponentially inside the solid with a decay length similar to the disturbance size. To examine the strain decay behavior, the phase along the dotted line in Figure 2b was extracted and the result is shown in Figure 2c. It is found that the phase change does follow an exponential decay, and by fitting the experimental data, the decay length can be derived, which is 50 ± 15 nm. Some other models can also be used to explain this strain relaxation behavior. For example, the ‘shear-lag’ approximation and the ‘lap shear’ model predict a hyperbolic cosine dependence on stress with respect to distance, which will also display an exponential decay near the edges.^129^ However, the distributed force model underestimates the elastic relaxation due to the free edges and does not show good agreement with the experiment results here.^137^ BCDI has the potential of being applied as a tool for distinguish between the different models of strain relaxation with nanoscale accuracy. Figure 2b and 2c also show that there are some positions in the film where the phases deviate from the base line; this may due to the presence of dislocations or misfit defects in the film during the epitaxial growth of strained Si on SiGe.

**Figure 13:**
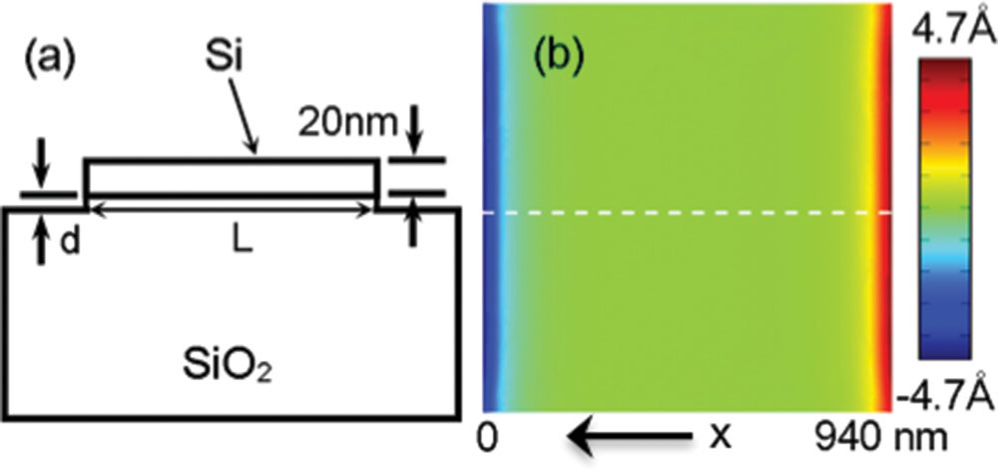
Schematic side view of the modeling system (a) and the simulated in-plane displacement in the x direction (b). Reproduced with permission.^136^ 2011, American Institute of Physics.

3D finite element analysis (FEA) was carried out using the COMSOL Multiphysics software, to gain more insight into the relaxation phenomenon. **Figure** **13**a is the schematic side view of the modelled system. It consists of a 20 nm thick L × L Si square on top of a 1 μm thick SiO_2_ layer and an additional thickness, *d*, of SiO_2_ underneath the structure to account for possible over-etching from the RIE process. Both materials have linear elasticity in the calculations. An initial tensile strain of 0.6% was applied to the Si layer. The relaxation phenomenon was then simulated by taking away the constraints at the free facets and allowing the system to achieve equilibrium. Simulation results show that without over-etching (*d* = 0 nm), the model predicts a too short relaxation decay length. Manual optimization of the model yields *L* = 940 ± 20 nm and *d* = 9 ± 3 nm.

Figure 3b shows the simulated in-plane displacement component along the *x* direction for *L* = 940 nm and *d* = 9 nm. It can be seen that the relaxations are pronounced near the edges of the square, in agreement with our BCDI experiment results. To compare with the experimentally measured phase changes, the displacements along the dotted line in Figure 3b are extracted and superposed in Figure 2c (red dashed line). It can be seen that they have very similar decay behaviours. The simulated displacement indeed follows an exponential decay from the edge and the fitted decay length is 45 nm, reasonably consistent with the experiment results. The above results demonstrate the ability of BCDI to probe the local strain on the nanoscale, as well as the morphological subtleties in semiconductor devices. It also shows that the magnitude of the strain relaxation in submicron and nano-scale sSOI structures will be significant and should be taken into consideration in the design and fabrication of sSOI-based devices. It is worth noting that the edge relaxation strain measured by BCDI is the local strain due to the patterning, while the initial tensile strain is global. The absolute strain of the patterned structure is the combination of the edge relaxation strain and the initial strain. As the initial biaxial tensile strain becomes relaxed, the band splitting and the resulting reduction in carrier average effective mass and intra-/inter- band scattering will not be as effective, and the carrier mobility enhancement will decay. It is also expected that the near oxide region becomes highly strained and near surface region can be fully relaxed.^138^ This non-uniform redistribution of strain on the nanoscale will impact on electrical and optical properties. Future work is planned to perform in situ BCDI measurements while operating the sSOI device, to directly correlate the strain distribution with the electrical performance figure-of-merit of the device.

One expected issue for measuring micro- or nano- scaled samples using synchrotron source is radiation damage. In our previous work, we observed that the Si nanowires patterned on SOI substrate started showing signs of radiation-induced damage when the total dose exceeded 1 × 10^10^ Gy (J·kg^−1^).^81^ In the current experiment, we measured the diffraction by rotating the sample over a range of 0.6° with a step size of 0.01°, and a 25 s exposure time at each step. This corresponds to a total exposure time of ∼25 min. The coherent flux of the beamline is approximately 10^9^ photons/s over the focal spot with an area of 1.5 μm^2^. One measurement would cause 1.5 × 10^12^ photons to be absorbed, amounting to a dose of 2.5 × 10^9^ Gy (J·kg^−1^). Compared with our previous work, it can be seen that the dose in this experiment is well within the safe range. We repeated the measurement a couple of times immediately after the first one, finding that both the diffraction pattern and the reconstructions remain unchanged. It is also worth pointing out that the presence of already existing defects will be the vulnerable part of the structure. For samples from the same material but with different existing fault states (for example, due to different process conditions), the properties associated with radiation damage will be different.^139^

#### 3.2.2 Strain Relaxation in Multiple sSOI Nanostructures

**Figure 14:**
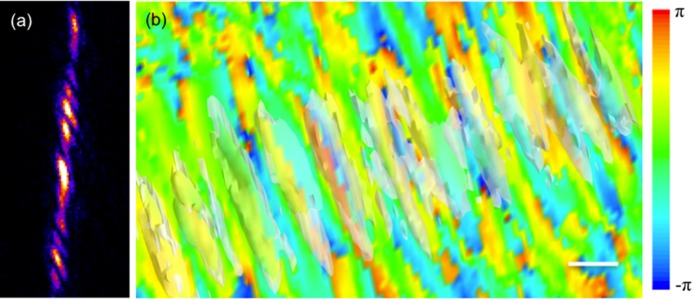
Diffraction pattern (central frame of the (–111) reflection) for an array of 11 sSOI wires (a); and the reconstructed phase map with the amplitude (translucent white) superimposed (b). scale bar = 300 nm

In a subsequent experiment, we attempted to measure strain effects in more complicated sSOI structures. sSOI wire arrays were fabricated using a sSOI substrate from similar wafers to those used for fabricating the above square sSOI structure, with the E-beam lithography and RIE etch patterning process. The array consists of 11 parallel wires with dimension of 100 nm × 1 μm and a 100 nm separation between them. The experiment geometry is the same as the one showing in Figure 10, with the long edges of the wires facing the incident beam. **Figure** **14**a shows the diffraction pattern (central frame along the (–111) Bragg reflection) from the wire array. The sample was measured at the 34-ID-C beamline, but with a more efficient “Medipix” detector, capable of photon counting over a 256 × 256 sensor array. Because the diffraction pattern extended quite far in the vertical direction, the detector is not large enough to collect the full pattern in this direction. The cut-off of high frequency signals limited the reconstruction resolution as will see in the following discussion.

Figure 4a appears to be a quite complicated pattern and the fringes are mostly along the vertical direction. This is due to the interference of the wires in the array. Figure 4b shows the reconstructed phase map, with the amplitude isosurface (shown in translucent white color) superimposed. There are 11 strips and each with a phase wrap along the wire horizontal edge direction. This is consistent with the strain relaxation behaviour due to the patterning process as seen in the square sSOI structure. However, the resolution achievable here was not high enough for resolving the relaxation inside each individual wire quantitatively. With improvement of beamline optics and detector techniques, higher quality data sets might be obtained to allow further detailed analysis for strain relaxation properties in more complicated structures that approach the industrial processing size scale.

## 4 Conclusion

BCDI has undergone rapid development since its first demonstration about a decade ago with respect to both the experimental method and the reconstruction algorithms. New applications in materials/nanoscience have been pursued. To date, most of the BCDI work has been conducted on third-generation synchrotron radiation sources, and the best spatial resolution achieved is around 10∼20 nm. The strain sensitivity, in the few pm range, has demonstrated great utility. With the advent of new coherent X-ray sources such as high harmonic generation and X-ray free-electron lasers, BCDI has the potential to be the method of choice for investigating sub-nanometer and atomic behaviour in three dimensions for nanomaterials, providing unprecedented insights into the strain distribution in semiconductor devices, functional materials under extreme conditions, as well as many fundamental processes such as diffusion, phase transitions, catalysis and imaging phonons (lattice dynamic) in nanocrystals. Its related research and applications will be even more exciting and rewarding in the coming decade.

## References

[1] Bartels RA, Paul A, Green H, Kapteyn HC, Murname MM, Backus S, Christov IP, Liu Yanwei, Attwood D, Jacobsen C (2002). Science.

[2] Takayama Y, Tai RZ, Hatano T, Miyhara T, Okamoto W, Kagoshima Y (1998). J. Synchrotron Radiat.

[3] van der Veen F, Pfeiffer F (2004). J. Phys. Condens. Matter.

[4] Vlieg E, de Vries SA, Alvarez J, Ferrer S (1997). J. Synchrotron Radiat.

[5] Bongaerts JHH, David C, Drakopoulos M, Zwanenburg MJ, Wegdam GH, Lackner T, Keymeulen H, vander Veen JF (2002). J. Synchrotron Radiat.

[6] Kohn V, Snigireva I, Snigirev A (2002). Phys. Rev. Lett.

[7] Sandy AR, Lurio LB, Mochrie SGJ, Malik A, Stephenson GB, Pelletier JF, Sutton M (1999). J. Synchrotron Radiat.

[8] Goodman JW (1985). Statistical Optics.

[9] Giannini C, Diaz A, Favre-Nicolin V, Guagliardi A, Masciocchi N (2010). Diffraction at the Nanoscale Nanocrystals, Defective and Amorphous Materials.

[10] Gerchberg RW, Saxton WO (1972). Optik.

[11] Miao J, Sayre D, Chapman HN (1998). J. Opt. Soc. Am. A.

[12] Fienup JR (1978). Opt. Lett.

[13] Fienup JR (1982). Appl. Optic.

[14] Bates RHT (1982). Optik.

[15] Fienup JR (1986). J. Opt. Soc. Am.

[16] Bruck YuM, Sodin LG (1979). Opt. Commun.

[17] Hayes MH (1982). IEEE Trans. Acoust., Speech, Signal Process.

[18] Fienup JR (1987). J. Opt. Soc. Am.

[19] Fienup JR, Wackerman CC (1986). J. Opt. Soc. Am. A.

[20] Williams GJ, Pfeifer MA, Vartanyants IA, Robinson IK (2007). Acta Crystallogr. A.

[21] Chen CC, Miao J, Wang CL, Lee TK (2007). Phys. Rev. B.

[22] Elser V (2003). J. Opt. Soc. Am. A.

[23] Elser V, Rankenburg I, Thibault P (2007). Proc. Natl. Acad. Sci. USA.

[24] Marchesini S (2007). J. Opt. Soc. Am. A.

[25] Bauschke HH, Combettes PL, Luke DR (2003). J. Opt. Soc. Am. A.

[26] Luke DR (2005). Inverse Probl.

[27] Oszlanyi G, Suto A (2008). Acta Crystallogr. Sect. A.

[28] Candes EJ, Eldar YC, Strohmer T, Voroninski V (2013). SIAM J. Imaging Sci.

[29] Marchesini S, He H, Chapman H, Hau-Riege S, Noy A, Howells M, Weierstall U, Spence J (2003). Phys. Rev. B.

[30] Miao JW, Charalambous P, Kirz J, Sayre D (1999). Nature.

[31] Miao JW, Johnson B, Anderson EH, Lai B, Hodgson KO (2002). Phys. Rev. Lett.

[32] Chapman HN, Barty A, Marchesini S, Noy A, Hau-Riege SP, Cui C, Howells MR, Rosen R, He H, Spence JC, Weierstall U, Beetz T, Jacobsen C, Shapiro D (2006). J. Opt. Soc. Am. A.

[33] Schroer CG, Boye P, Feldkamp JM, Patomme J, Schropp A, Schwab A, Stephan S, Burghammer M, Schöder S, Riekel C (2008). Phys. Rev. Lett.

[34] Takahashi Y, Nishino Y, Tsutsumi R, Furukawa H, Mimura H, Zettsu N, Matsubara E, Ishikawa T, Yamauchi K (2009). Phys. Rev. B.

[35] Barty A, Marchesini S, Chapman HN, Noy A, Hau-Riege SR, Cui C, Howells MR, Shapiro DA, Minor AM, Spence JCH, Weierstall U, Ilavsky J, Noy A, Hau-Riege SP, Artyukhin AB, Baumann T, Willey T, Stolken J, van Buuren T, Kinney JH (2008). Phys. Rev. Lett.

[36] Jiang H, Xu R, Chen C, Yang W, Fan J, Tao X, Song C, Kohmura Y, Xiao T, Wang Y, Fei Y, Ishikawa T, Mao W, Miao J (2013). Phys. Rev. Lett.

[37] Williams GJ, Quiney HM, Dhal BB, Tran CQ, Nugent KA, Peele AG, Paterson D, de Jonge MD (2006). Phys. Rev. Lett.

[38] Quiney HM, Peele AG, Cai Z, Paterson D, Nugent KA (2006). Nat. Phys.

[39] De Caro L, Giannini C, Pelliccia D, Mocuta C, Metzger TH, Guagliardi A, Cedola A, Burkeeva I, Lagomarsino S (2008). Phys. Rev. B.

[40] Quiney HM, Nugent KA, Peele AG (2005). Opt. Lett.

[41] Xiao X, Shen Q (2005). Phys. Rev. B.

[42] Nugent KA (2010). Adv. Phys.

[43] Abbey B, Nugent KA, Williams GJ, Clark JN, Peele AG, Pfeifer MA, de Jonge M, McNulty I (2008). Nat. Phys.

[44] Abbey B, Williams GJ, Pfeifer MA, Clark JN, Putkunz CT, Torrance A, McNulty I, Levin TM, Peele AG, Nugent KA (2008). Appl. Phys. Lett.

[45] Peterson I, Abbey B, Putkunz CT, Vine DJ, van Riessen GA, Cadenazzi GA, Balaur E, Ryan R, Quiney HM, McNulty I, Peele AG, Nugent KA (2012). Opt. Express.

[46] Jones MWM, van Riessen GA, Abbey B, Putkunz CT, Junker MD, Balaur E, Vine DJ, McNulty I (2013). Sci. Rep.

[47] Hoppe W (1969). Acta Cryst. A.

[48] Faulkner HML, Rodenburg JM (2004). Phys. Rev. Lett.

[49] Rodenburg JM, Faulkner HML (2004). Appl. Phys. Lett.

[50] Thibault P, Dierolf M, Menzel A, bunk O, David C, Pfeiffer F (2008). Science.

[51] Schropp A, Boye P, Feldkamp JM, Hoppe R, Patommel J, Samberg D (2010). Appl. Phys. Lett.

[52] de la Cuesta FB, Wenger MPE, Bean RJ, Horton MA, Robinson IK (2009). Proc. Natl. Acad. Sci. USA.

[53] Robinson IK, Vartanyants IA, Williams GJ, Pfeifer MA, Pitney JA (2001). Phys. Rev. Lett.

[54] Williams GJ, Pfeifer MA, Vartanyants IA, Robinson IK (2003). Phys. Rev. Lett.

[55] Pfeifer MA, Williams GJ, Vartanyants IA, Harder R, Robinson IK (2006). Nature.

[56] Robinson I, Harder R (2009). Nat. Mater.

[57] Takagi S (1969). J. Phys. Soc. Jpn.

[58] Lemaitre J, Chaboche JL (1994). Mechanics of Solid Materials.

[59] Newton MC, Leake SJ, Harder R, Robinson IK (2010). Nat. Mater.

[60] Clark JN, Huang X, Harder R, Robinson IK (2012). Nat. Commun.

[61] Robinson IK, Vartanyants IA, Williams GJ, Pfeifer MA, Pitney JA (2001). Phys. Rev. Lett.

[62] Vartanyants I, Robinson I (2001). J. Phys.

[63] Leake SJ, Newton MC, Harder R, Robinson IK (2009). Opt. Express.

[64] Williams GJ, Quiney HM, Peele AG, Nugent KA (2007). Phys. Rev. B.

[65] Huang WJ, Zuo JM, Jiang B, Kwon KW, Shim M (2009). Nat. Phys.

[66] Vartanyants IA, Singer A (2010). New J. Phys.

[67] Whitehead LW, Williams GH, Quiney HM, Vine DJ, Dilanian RA, Flewett S, Nugent KA, Peele AG, Balaur E, McNulty I (2009). Phys. Rev. Lett.

[68] Clark JN, Peele AG (2011). Appl. Phys. Lett.

[69] Chen B, Dilanian RA, Teichmann S, Chen B, Dilanian RA, Teichmann S, Abbey B, Peele AG, Williams GJ, Hannaford P, Van Dao L, Quiney HM, Nugent KA (2009). Phys. Rev. A.

[70] Abbey B, Whitehead LW, Quiney HM, Vine DJ, Cadenazzi GA, Henderson CA, Nugent KA, Balaur E, Putkunz CT, Peele AG, Williams GJ, McNulty I (2011). Nat. Photon.

[71] Harder R, Liang M, Sun Y, Xia Y, Robinson IK (2010). New J. Phys.

[72] Watari M, McKendry R, Voegtli M, Aeppli G, Soh Y-A, Shi X (2011).

[73] Gulden J, Yefanov OM, Mancuso AP, Singer A, Bernátová V, Burkhardt A, Polozhentsev O, Soldatov A, Sprung M, Vartanyants IA (2012). Opt. Express.

[74] Robinson Ian (2013). J. Phys. Soc. Jpn.

[75] Beutier G, Verdier M, Parry G, Gilles B, Labat S, Richard M-I, Cornelius T, Lory P-F, Hoang SV, Livet F, Thomas O, De Boissieu M (2013). Thin Solid Films.

[76] Harder R, Robinson I (2013). J. Microsc.

[77] Favre-Nicolin NV, Mastropietro F, Eymery J, Camacho D, Niquet YM, Borg BM, Messing ME, Wernersson L-E, Algra RE, Bakkers EPAM, Metzger TH, Harder R, Robinson IK (2010). New J. Phys.

[78] Leake SJ, Harder R, Robinson IK (2011). New J. Phys.

[79] Xiong G, Huang X, Leake S, Newton MC, Harder R, Robinson IK (2011). New J. Phys.

[80] Minkevich AA, Fohtung E, Slobodskyy T, Riotte M, Grigoriev D, Metzger T, Irvine AC, Novak V, Holy V, Baumbach T (2011). Europhys. Lett.

[81] Shi X, Xiong G, Huang X, Harder R, Robinson I (2012). New J. Phys.

[82] Haag ST, Richard M, Welzel U, Favre-Nicoli V, Balmes O, Richter G, Mittemeijer EJ, Thomas O (2013). Nano Lett.

[83] Cha W, Song S, Jeong NC, Harder R, Yoon KB, Robinson IK, Kim H (2010). New J. Phys.

[84] Cha W, Jeong N, Song S, Park H, Pham T, Harder R, Lim B, Xiong G, Ahn D, McNulty I, Kim J, Yoon K, Robinson IK, Kim H (2013). Nat. Mater.

[85] Yang W, Huang X, Harder R, Clark JN, Robinson IK, Mao H (2013). Nat. Commun.

[86] Clark J, Beitra L, Xiong G, Higginbotham A, Fritz DM, Lemke HT, Zhu D, Chollet M, Williams GJ, Messerschmidt M, Abbey B, Harder RJ, Korsunsky AM, Wark JS, Robinson IK (2013). Science.

[87] Tanaka Y, Ito K, Nakatani T, Onitsuka R, Newton M, Sato T, Togashi T, Yabashi M, Kawaguchi T, Shimada K, Tokuda K, Takahashi I, Ichitsubo T, Matsubura E, Noshino Y (2013). J. Ceram. Soc. Jpn.

[88] Xiong G, Clark JN, Nicklin C, Rawle J, Robinson IK

[89] Boutet S, Robinson IK (2008). J. Synchrotron Radiat.

[90] Aranda MAG, Berenguer F, Bean RJ, Shi X, Xiong G, Collins SP, Nave C, Robinson IK (2010). J. Synchrotron Radiat.

[91] Newton MC, Harder R, Huang X, Xiong G, Robinson IK (2010). Phys. Rev. B.

[92] Vaxelaire N, Proudhon H, Labat S, Kirchlechner C, Keckes J, Jacques V, Ravy S, Forest S, Thomas O (2010). New J. Phys.

[93] Shi X, Xiong G, Huang X, Harder R, Robinson I (2010). Z. Kristalogr.

[94] Jacques VLR, Ravy S, Le Bolloc'h D, Pinsolle E, Sauvage-Simkin M, Livet F (2011). Phys. Rev. Lett.

[95] Huang X, Harder R, Xiong G, Shi X, Robinson I (2011). Phys. Rev. B.

[96] Chen B, Zhang F, Berenguer F, Bean R, Kewish C, Vila-Comamala J, Chu Y, Rodenburg J, Robinson I (2011). New J. Phys.

[97] Huang X, Harder R, Leake S, Clark J, Robinson I (2012). J. Appl. Crystallogr.

[98] Pinsolle E, Kirova N, Jacques VLR, Sinchenko AA, LeBolloc'h D (2012). Phys. Rev. Lett.

[99] Moore GE (1965). Electronics.

[100] International Technology Roadmap for Semiconductors 2012, http://public.itrs.net.

[101] Colinge JP (1997). Silicon-on-Insulator Technology: Materials to VLSI.

[102] Celler GK (1994). Silicon-on-Insulator Structures: Fabrication, The Encyclopedia of Advanced Materials.

[103] Pelloie JL, Auberton-Herve A (2001). Solid State Technol.

[104] Vandooren A, Jovanovic D, Egley S, Sadd M, Nguyen BY, White B, Orlowski M, Mogab J (2002). Proc. IEEE Int. SOI Conf. Williamsburg.

[105] Fossum JG, Trivedi VP, Wu K (2002). Proc. IEEE Int. SOI Conf. Williamsburg.

[106] Celler CK, Cristoloveanu S (2003). J. Appl. Phys.

[107] Rudenko T, Kilchytska V, Colinge JP, Dessard V, Flandre D (2001). IEEE Electr. Device Lett.

[108] Langdo TA, Lochtefeld A, Currie MT, Hammond R, Yang VK, Carlin JA, Vinei CJ (2002). Proc. IEEE Int. SOI Conf. Williamsburg.

[109] Reiche M, Moutanabbir O, Himcinschi C, Christiansen SH, Erfurth W, Gosele U, Mantl S, Buca D, Zhao QT, Loo R, Nguyen D, Muster F, Petzold M (2008). ECS Trans.

[110] Sun G, Sun Y, Nishida T, Thompson SE (2007). J. Appl. Phys.

[111] Suthram S, Ziegert JC, Nishida T, Thompson SE (2007). IEEE Elec. Dev. Lett.

[112] Leong M, Doris B, Kedzierski J, Rim K, Yang M (2004). Science.

[113] Rahman A, Lundstrom MS, Ghosh AW (2005). J. Appl. Phys.

[114] Sun Y, Thompson SE, Nishida T (2007). J. Appl. Phys.

[115] Lundstrom M (2000). Fundamentals of Carrier Transport.

[116] Shimizu A, Hachimine K, Ohki N, Ohta H, Koguchi M, Nonaka Y, Sato H, Ootsuka F (2001). Int. Elec. Dev. Meet.

[117] Gannavaram S, Pesovic N, Ozturk MC (2000). Int. Elec. Dev. Meet.

[118] Tarun A, Hayazawa N, Ishitobi H, Kawata S, Reiche M, Moutanabbir O (2011). Nano Lett.

[119] Moutanabbir O, Reiche M, Hahnel A, Erfurth W, Gosele U, Motohashi M, Tarun A, Hayazawa N, Kawata S (2010). Nanotechnology.

[120] Koester SJ, Rim K, Chu JO, Mooney PM, Ott JA, Hargrove MA (2001). Appl. Phys. Lett.

[121] Lei RZ, Tsai W, Aberg I, O'Reilly TB, Hoyt JL, Antoniadis DA, Smith HI, Paul AJ, Green ML, Li J, Hull R (2005). Appl. Phys. Lett.

[122] Himcinschi C, Singh R, Radu I, Milenin AP, Erfurth W, Reiche M, Gösele U, Christiansen SH, Muster F, Petzold M (2007). Appl. Phys. Lett.

[123] Jain SC, Maes HE, Pinardi K, De Wolf I (1996). J. Appl. Phys.

[124] Moutanabbir O, Reiche M, Hähnel A, Erfurth W, Motohashi M, Tarun A, Hayazawa N, Kawata S (2010). Appl. Phys. Lett.

[125] Tarun A, Hayazawa1 N, Balois MV, Kawata S, Reiche M, Moutanabbir O (2013). New J. Phys.

[126] Armigliato A, Balboni R, Carnevale GP, Pavia G, Piccolo D, Frabboni S, Benedetti A, Cullis AG (2003). Appl. Phys. Lett.

[127] Cooper D, Béché A, Hartmann J-M, Carron V, Rouvie're J-L (2010). Appl. Phys. Lett.

[128] Hähnel A, Reiche M, Moutanabbir O, Blimtritt H, Geisler H, Hoentschel J, Engelmann HJ (2012). Microsc. Microanal.

[129] Murray CE, Yan HF, Noyan IC, Cai Z, Lai B (2005). J. Appl. Phys.

[130] Murray CE, Polvino SM, Noyan IC, Cai Z, Maser J, Holt M (2013). Thin Solid Films.

[131] Cohen GM, Mooney PM, Jones EC, Chan KK, Solomon PM, Wong HSP (1999). Appl. Phys. Lett.

[132] Gailhanou M, Loubens A, Micha JS, Charlet B, Minkevich AA, Fortunier R, Thomas O (2007). Appl. Phys. Lett.

[133] Baumbach T, Lübbert D (1999). J. Phys. D.

[134] Godard P, Carbone G, Allain M, Mastropietro F, Chen G, Capello L, Diaz A, Metzger TH, Stangl J, Chamard V (2011). Nat. Commun.

[135] Hruszkewycz SO, Holt MV, Murray CE, Bruley J, Holt J, Tripathi A, Shpyrko OG, McNulty I, Highland MJ, Fuoss PH (2012). Nano Lett.

[136] Xiong G, Moutanabbir O, Huang X, Paknejad SA, Shi X, Harder R, Reiche M, Robinson IK (2011). Appl. Phys. Lett.

[137] Hu SM (1979). J. Appl. Phys.

[138] Moutanabbir O, Reiche M, Hahnel A, Oehme M, Kasper E (2010). Appl. Phys. Lett.

[139] Favre-Nicolin V, Eymery J, Koester R, Gentile P (2009). Phys. Rev. B.

